# Penalized Exponentially Tilted Likelihood for Growing Dimensional Models with Missing Data

**DOI:** 10.3390/e27020146

**Published:** 2025-02-01

**Authors:** Xiaoming Sha, Puying Zhao, Niansheng Tang

**Affiliations:** Yunnan Key Laboratory of Statistical Modeling and Data Analysis, Yunnan University, Kunming 650050, China; xmsha@mail.ynu.edu.cn (X.S.); pyzhao@ynu.edu.cn (P.Z.)

**Keywords:** estimating equations, growing dimensional models, missing at random, penalized exponentially tilted likelihood, Wilks’ property

## Abstract

This paper develops a penalized exponentially tilted (ET) likelihood to simultaneously estimate unknown parameters and select variables for growing dimensional models with missing response at random. The inverse probability weighted approach is employed to compensate for missing information and to ensure the consistency of parameter estimators. Based on the penalized ET likelihood, we construct an ET likelihood ratio statistic to test the contrast hypothesis of parameters. Under some wild conditions, we obtain the consistency, asymptotic properties, and oracle properties of parameter estimators and show that the constrained penalized ET likelihood ratio statistic for testing the contrast hypothesis possesses the Wilks’ property. Simulation studies are conducted to validate the finite sample performance of the proposed methodologies. Thyroid data taken from the First People’s Hospital of Yunnan Province is employed to illustrate the proposed methodologies.

## 1. Introduction

Estimating equations (EEs) are widely used to make statistical inference on data with unknown distribution but moment conditions. Generally, it can be formulated as $g\left(x,y;\beta \right)=(g_{1}\left(x,y;\beta \right),g_{2}\left(x,y;\beta \right),\ldots ,g_{r}\left(x,y;\beta \right))$, satisfying the following moment restrictions, E[g(x,y;β)]=0, where {x,y} are response variables or covariates and β is an unknown *p*-dimensional parameter vector. There is considerable literature on the statistical inference of EEs. For example, Godambe [1] discussed the properties (e.g., unbiasedness) of unknown parameter estimators in EEs. Carroll et al. [2] applied EEs to measurement error models. Hardin & Hilbe [3] extended EEs to a more general case. For the fixed and finite values of *p* and *r*, various methods have been developed to make statistical inference on EEs. For example, see the generalized method of moments (GMM) [4], the empirical likelihood (EL) method [5,6,7], and the exponentially tilted (ET) likelihood method [8]. In particular, Newey & Smith [9] showed that EL- and ET-likelihood-based estimators of unknown parameters in EEs belong to a class of generalized EL (GEL) estimators. The GEL estimator, as an alternative to the GMM estimator, possesses the Wilks’ property, Bartlett correction, and well-defined high-order asymptotic properties, which are superior to GMM estimation [9,10].

Estimating equations for growing dimensional models has extensive applications in statistics, economics, finance, and other related fields. When *p* and *r* grow to infinity, it usually assumes that these models are sparse, meaning that only a small number of covariates contribute to the response variable. Hence, many penalized approaches have been developed to simultaneously estimate unknown parameters and select important covariates. Introducing penalty functions into likelihood is a widely adopted approach; there are many choices for penalty functions, such as least absolute shrinkage and selection operator (Lasso) [11], adaptive Lasso [12], group Lasso, smoothly clipped absolute deviation (SCAD) [13], and folded concave penalty (FCP) [14], among others. For example, Hastie et al. [15] showed that introducing penalty functions into likelihood can adjust the trade-off between bias and variance. Lam & Fan [16] studied the profile-kernel likelihood method in linear regression models. Zou & Zhang [17] established the oracle property of the adaptive elastic-net under the divergent parameter model. Caner & Zhang [18] extended Zou & Zhang’s work to GMMs. Tang et al. [8] proposed an exponentially tilted (ET) likelihood inference for EEs with growing dimensional models, studied the asymptotic properties of ET likelihood estimators, and showed that the ET likelihood estimators of unknown parameters are robust enough to model misspecification and the ET likelihood ratio statistic for testing parameters and possess the Wilks’ property under some wild conditions. The aforementioned literature only focuses on situations where the data are completely observed. However, missing data are widely encountered in many applications, such as biomedical studies and clinical trials, due to various reasons, such as data collection errors, non-response, or technical limitations.

In data analysis, the imputation of missing data is critical when the missing data mechanism is not missing completely at random, in that the complete case analysis, which is conducted using only the observations with completely observed variables, may lead to severe bias. To address the issue, many methods have been developed to make statistical inference on missing data models via the imputation of missing data in various fields, including economics, social sciences, healthcare, and machine learning. For example, Wang et al. [19] investigated the estimation problem of regression coefficients in linear models with missing covariates. Ref. [20] applied locally weighted kernel polynomial regression methods to generalized linear models with missing predictors. Scharfstein et al. [21] studied the nonignorable drop-out problem in semiparametric nonresponse models. FitzGerald [22] proposed a weighted approach to addressing missing data in generalized estimating equations with binary outcomes. Wang et al. [23] presented a general approach to handling missing data in a longitudinal study based on expected EEs. Zhou et al. [24] redefined the EE imputed method using the kernel regression method to mitigate the biased effects of missing data. Tang et al. [25] developed an EL inference on parameters in generalized EEs with nonignorable missing response data. Tang et al. [8] proposed a nonparametric imputation method based on the propensity score in a general class of semiparametric models for missing data. Qi et al. [26] reformulated the EE imputed method via the kernel regression method to handle the problem of nonignorable missing data and utilized GMM to simultaneously estimate model parameters and tilting parameters in propensity score functions. Shao & Wang [27] investigated the nonignorable missing data with a semiparametric exponential tilting propensity and applied the inverse propensity weighting approach to estimating population parameters. Tang et al. [28] proposed a new feature screening procedure in ultrahigh-dimensional partially linear models with missing responses at random for longitudinal data based on the profile marginal kernel-assisted EE imputation technique. Liu and Yuan [29] proposed adaptive EL estimators to improve the estimation efficiency of existing adaptive estimators of the mean response with nonignorable nonresponse data. The aforementioned literature on the imputation of missing data mainly focus on EL inference for fixed-dimensional EEs. Although Tang et al. [8] considered an ET likelihood inference on EEs with growing dimensional models to address the shortcoming of EL inference that is not robust to the misspecification of EEs, to our knowledge, there is little work on ET likelihood inference on growing dimensional models with missing responses at random.

In this paper, we conduct statistical inference on growing dimensional models with missing data and propose a penalized ET likelihood to simultaneously estimate parameters and select variables. We consider the case where data including response variables and covariates are subject to missing at random, impute information for missing data via the inverse probability weighted (IPW) approach, and develop the ET likelihood estimators of parameters based on the EE imputed method together with penalized ET likelihood. When the penalty function satisfies certain conditions, we obtain the oracle property and asymptotic properties of parameter estimators and show that the constrained penalized ET likelihood ratio statistic for testing the contrast hypothesis asymptotically follows the centred chi-squared distribution. Based on the test statistics, we construct confidence intervals of parameters. We conduct simulation studies for several settings to verify the finite sample performance of the proposed methodologies, including the robustness and oracle properties of parameter estimators. Thyroid data taken from the First People’s Hospital of Yunnan Province is used to select important variables associated with the thyroid nodules disease.

The rest of this paper is organized as follows. Section 2 constructs the penalized ET likelihood and develops the ET likelihood estimators of parameters and the penalized ET likelihood ratio statistic for testing te contrast hypothesis. Section 3 studies the oracle properties and asymptotic properties of parameter estimators and shows the Wilks’ property of the asymptotic distribution of the constrained penalized ET likelihood ratio statistic for testing the contrast hypothesis under some regularization conditions. Section 4 conducts simulation studies to assess the finite sample performance of the proposed methodologies. An example is illustrated in Section 5. A brief discussion is given in Section 6.

## 2. Model and Notation

Let ${\left\{X_{i},Y_{i}\right\}}_{i=1}^{n}$ be *n* independent and identically distributed observations of random variables {X,Y} coming from an unknown distribution F(x,y), where $X_{i}$’s are the $d_{x}$-dimensional vector and $Y_{i}$’s are the $d_{y}$-dimensional vector. It is assumed that $X_{i}$’s are always observed, while $Y_{i}$’s are subject to missingness for i=1,…,n. Let $\delta_{i}$ be an indicator of $Y_{i}$, i.e., $\delta_{i}=1$ if $Y_{i}$ is observed and $\delta_{i}=0$ if $Y_{i}$ is missing for i=1,…,n. It is assumed that $\delta_{i}$ and $\delta_{j}$ are independent for any i≠j. Here, we assume a missing at random (MAR) mechanism for missing value of $Y_{i}$, i.e., $\delta_{i}$ only depends on $X_{i}$, such that $\mathrm{Pr}\left(\delta_{i}=1|X_{i}\right)≐\pi_{i}\left(X_{i};\gamma \right)$ for i=1,…,n, where γ is a *q*-dimensional unknown parameter vector.

To meet the identifiability condition of the considered model, the idea of instrumental variables [30] is adopted. Thus, it is assumed that $X_{i}$ can be decomposed into $X_{i}={\left(U_{i}^{⊤},Z_{i}^{⊤}\right)}^{⊤}$, where $Z_{i}$ is a $d_{z}$-dimensional vector of instrumental variables. Under the above assumption, the missingness data mechanism model can be expressed as $\mathrm{Pr}\left(\delta_{i}=1|X_{i}\right)=\mathrm{Pr}\left(\delta_{i}=1|U_{i}\right)=\pi_{i}\left(U_{i};\gamma \right)$, which can be formulated by a logistic regression. That is,

(1) $$\begin{array}{c} \delta_{i}|U_{i}∼B\left(\pi_{i}\left(U_{i};\gamma \right)\right) \end{array}$$

where B(a) represents the Bernoulli distribution with the success probability *a*,

(2) $$\begin{array}{c} \mathrm{logit}\left\{\pi_{i}\left(U_{i};\gamma \right)\right\}=\gamma_{c}+U_{i}^{⊤}\gamma_{u}, \end{array}$$

where logit(a)=log{a/(1−a)}, $\gamma =\left(\gamma_{c},\gamma_{u}^{⊤}\right)={\left(\gamma_{1},\ldots ,\gamma_{q}\right)}^{⊤}$ is the *q*-dimensional vector of unknown parameters associated with propensity score function $\pi_{i}\left(U_{i};\gamma \right)$. To estimate unknown parameters, we consider the following calibration conditions:

(3) $$\begin{array}{c} \varphi \left(U_{i},Z_{i};\gamma \right)=\left\{\frac{\delta_{i}}{\pi_{i}\left(U_{i};\gamma \right)}-1\right\}d\left(Z_{i}\right),\;i=1,2,\ldots ,n, \end{array}$$

where $d(Z_{i})$ is any users-specified function or vector on $Z_{i}$. If $\pi_{i}\left(U_{i};\gamma \right)$ is correctly specified, we have $E[\varphi \left(U_{i},Z_{i};\gamma \right)]=0$. Stock [31] discussed the conditions for the valid instrumental variables, along with some common pitfalls on the applications of instrumental variables.

Let $\beta_{0}$ be a *p*-dimensional vector of true parameters satisfying $E\{g\left(X_{i},Y_{i};\beta_{0}\right)\}=0$. Here, $g\left(X,Y;\beta \right)={\left(g_{1}\left(X,Y;\beta \right),\ldots ,g_{r}\left(X,Y;\beta \right)\right)}^{⊤}$ represents *r* unconditional moment restrictions with r≥p. To estimate parameter vector γ associated with the missingness data mechanism, we assume that $\varphi \left(U_{i},Z_{i};\gamma \right)={\left(\varphi_{1}\left(U_{i},Z_{i};\gamma \right),\ldots ,\varphi_{m}\left(U_{i},Z_{i};\gamma \right)\right)}^{⊤}$ satisfies $E\{\varphi \left(U_{i},Z_{i};\gamma_{0}\right)\}=0$ for i=1,…,n, where $\gamma_{0}$ is the true values of parameter γ. Our key purpose is to make statistical inference on β and γ for growing dimensional estimating equations and unknown parameters, i.e., {r,m,p,q} are allowed to diverge with the sample size *n*.

To make statistical inference on unknown parameter vectors β and γ, we consider the inverse probability weighted (IPW) method, which is an important method for handling missing data and has been widely applied to various missing data analyses. For example, Rosenbaum & Rubin [32] provided a detailed argument on the application of propensity scores for adjusting weights and reducing the bias of selection. Imai & Ratkovic [33] developed the IPW method to estimate causal effects. Robins et al. [34] proposed a semiparametric approach based on inverse probability weighted EEs to handle missing data. Robins & Rotnitzky [35] discussed the application of the IPW method for improving parameter estimation efficiency in the presence of missing data. Hirano et al. [36] applied the IPW method to efficiently estimate the average effect of treatments in the presence of missing data. Inspired by the aforementioned literature, we consider the following IPW-based EEs:

(4) $$\begin{array}{c} g^{ipw}\left(X_{i},Y_{i},U_{i};\beta ,\gamma \right)=\frac{\delta_{i}}{\pi_{i}\left(U_{i};\gamma \right)}g\left(X_{i},Y_{i};\beta \right). \end{array}$$

Combining the calibration conditions (3) and (4) leads to the following modified estimating equations:

(5) $$\begin{array}{c} G\left(D_{i};\beta ,\gamma \right)=\left(g_{1}^{ipw}\left(D_{i};\beta ,\gamma \right),\ldots ,g_{r}^{ipw}\left(D_{i};\beta ,\gamma \right),\varphi_{1}\left(U_{i};\gamma \right),\ldots ,\varphi_{m}\left(U_{i};\gamma \right)\right), \end{array}$$

where $D_{i}=\left\{X_{i},Y_{i},U_{i}\right\}$ for i=1,…,n.

Let t=r+m represent the dimension of the modified estimating equations and $\theta ={\left(\beta^{⊤},\gamma^{⊤}\right)}^{⊤}$ be an *s*-dimensional vector of the parameters associated with the modified estimating equations, where s=p+q. When t≥s, the estimators of parameters θ can be directly obtained by solving estimating Equation (5), but this is not necessarily feasible, especially when t>s, i.e., the number of estimating equations is greater than the number of parameters, implying that estimating equations provide external available information, which may lead to the over-identification problem. Hansen [4] showed that this external available information can improve estimation efficiency in the presence of over-identification. Due to the fact that the ET likelihood is more robust than the EL likelihood in the presence of model misclassification [8], here we utilize the ET likelihood method to estimate parameters θ. Following the literature [8], the ET likelihood for θ based on the data $\left\{D_{i}:i=1,\ldots ,n\right\}$ can be defined as

(6) $$\begin{array}{c} L\left(\theta \right)=\inf\left\{\sum_{i=1}^{n}w_{i}\log\left(w_{i}\right):w_{i}\geq 0,\sum_{i=1}^{n}w_{i}=1,\sum_{i=1}^{n}w_{i}G\left(D_{i};\theta \right)=0\right\}. \end{array}$$

With the Lagrange multiplier method, we obtain

$$w_{i}=\frac{\exp\{\lambda^{⊤}G\left(D_{i};\theta \right)\}}{\sum_{j=1}^{n}\exp\left\{\lambda^{⊤}G\left(D_{j};\theta \right)\right\}},$$

where λ is the Lagrange multiplier. Also, the global minimizer of $w_{i}$ is reached when $w_{i}=n^{-1}$ when there are no constrained conditions. Thus, the profiled log-ET likelihood ratio function of θ based on the data $\left\{D_{i}:i=1,\ldots ,n\right\}$ can be expressed as

(7) $$\begin{array}{c} l_{n}\left(\lambda ,\theta \right)=-\left\{L\left(\theta \right)+\log\left(n\right)\right\}=\log\left[\frac{1}{n}\sum_{i=1}^{n}\exp\left\{\lambda^{⊤}G\left(D_{i};\theta \right)\right\}\right]. \end{array}$$

Let

$$\begin{array}{cc} U_{n1}\left(\lambda ,\theta \right)= & \partial l_{n}\left(\lambda ,\theta \right)/\partial \lambda =n^{-1}\sum_{i=1}^{n}\exp\left\{\lambda^{⊤}G\left(D_{i};\theta \right)\right\}G\left(D_{i};\theta \right),\\ U_{n2}\left(\lambda ,\theta \right)= & \partial l_{n}\left(\lambda ,\theta \right)/\partial \theta =n^{-1}\sum_{i=1}^{n}\exp\left\{\lambda^{⊤}G\left(D_{i};\theta \right)\right\}\lambda^{⊤}\left\{\partial G\left(D_{i};\theta \right)/\partial \theta \right\}, \end{array}$$

and

$$\begin{array}{c} {\hat{\theta }}^{\mathrm{ET}}=\arg\max_{\theta \in \Theta }\underset{\lambda \in {\hat{\Lambda }}_{n}\left(\theta \right)}{\inf}l_{n}\left(\lambda ,\theta \right), \end{array}$$

where ${\hat{\Lambda }}_{n}\left(\theta \right)=\left\{\lambda :\lambda^{⊤}G\left(D_{i};\theta \right)\in \varepsilon ,i=1,\ldots ,n\right\}$, in which ε is an open ball containing zero. Under some regularity conditions, ${\hat{\theta }}^{\mathrm{ET}}$ can be obtained by simultaneously solving $U_{n1}\left(\lambda ,\theta \right)=0$ and $U_{n2}\left(\lambda ,\theta \right)=0$, and the Lagrange multiplier λ satisfies $U_{n1}\left(\lambda ,{\hat{\theta }}^{\mathrm{ET}}\right)=0$.

In growing dimensional estimating equations, there is a well-known ill-posed problem [8]. To address this issue, we impose a sparsity assumption on parameter vectors θ and γ so that only a very small portion of their coordinates are non-zero, which means only a small number of covariates contribute to the response variable. Under the sparsity assumption, let $A_{\beta }=\left\{j:\beta_{0j}\neq 0\right\}$ and $d_{\beta }=\left|A_{\beta }\right|$ be the cardinality of $A_{\beta }$, where $\beta_{0j}$ represents the *j*th component of the true value $\beta_{0}$ of parameter vector β. Without loss of generality, let $\beta ={\left(\beta_{1}^{⊤},\beta_{2}^{⊤}\right)}^{⊤}$, where $\beta_{1}\in R^{d_{\beta }}$ and $\beta_{2}\in R^{p-d_{\beta }}$ correspond to the non-zero and zero components of β, respectively. Similarly, let $A_{\gamma }=\left\{j:\gamma_{0j}\neq 0\right\}$, its cardinality be $d_{\gamma }=\left|A_{\gamma }\right|$, and $\gamma ={\left(\gamma_{1}^{⊤},\gamma_{2}^{⊤}\right)}^{⊤}$, where $\gamma_{1}\in R^{d_{\gamma }}$ and $\gamma_{2}\in R^{q-d_{\gamma }}$ correspond to non-zero and zero components of γ, respectively. Also, let $A_{\theta }=\left\{j:\theta_{0j}\neq 0\right\}$, $d_{\theta }=\left|A_{\theta }\right|$, $\theta ={\left(\theta_{1}^{⊤},\theta_{2}^{⊤}\right)}^{⊤}$, where $\theta_{1}\in R^{d_{\theta }}$ and $\theta_{2}\in R^{s-d_{\theta }}$, where $d_{\theta }=d_{\beta }+d_{\gamma }$. Here, we consider simultaneously selecting non-zero components of both parameters of γ associated with the missingness data mechanism and parameter β related to growing dimensional EEs. To this end, we define the following penalized log-ET likelihood ratio function of θ based on the data $\left\{D_{i}:i=1,\ldots ,n\right\}$:

(8) $$\begin{array}{c} l_{pn}\left(\theta \right)=\log\left[\frac{1}{n}\sum_{i=1}^{n}\exp\left\{\lambda^{⊤}G\left(D_{i};\theta \right)\right\}\right]-\sum_{j=1}^{p}p_{\nu_{1}}\left(\right|\beta_{j}\left|\right)-\sum_{k=1}^{q}p_{\nu_{2}}\left(\right|\gamma_{k}\left|\right), \end{array}$$

where $p_{\nu }\left(\cdot \right)$ is some proper penalty function, with the tuning parameter ν controlling the trade-off between bias and model complexity. There are many choices for penalty functions, such as Lasso, adaptive Lasso, SCAD, FCP, and MCP. When the penalty function is non-convex, the calculation of the penalized ET likelihood estimation of parameters θ is challenging [37]. In particular, for the SCAD penalty function, the nested optimization algorithm is adopted to minimize the objective function $l_{pn}\left(\theta \right)$ given in (8) by using the local quadratic approximation method of Fan & Li [13] to approximate the nonconvex penalty $p_{\nu }\left(\right|\theta_{j}\left|\right)$ for j=1,…,p+q. The computational complexity of optimizing Equation (8) is $O(nts^{3}T)$, where T is the number of iterations.

For the selection of tuning parameter $\nu_{k}$ (k=1,2), there are many widely used methods, such as Akaike information criterion (AIC), Bayesian information criterion (BIC), cross validation (CV), and generalized CV, among others. Similarly to [8], we consider the Bayesian information criterion,

$$\begin{array}{c} \mathrm{BIC}\left(\nu \right)=-2l\left({\hat{\theta }}_{\nu }\right)+B_{n}\log\left(n\right)df_{\nu } \end{array}$$

to select the tuning parameter $\nu_{k}$, where $\nu =\{\nu_{1},\nu_{2}\}$, $l\left(\theta \right)=l_{n}\left(\lambda ,\theta \right)$ is given in Equation (7), ${\hat{\theta }}_{\nu }$ is the penalized ET estimator of θ depending on the tuning parameter ν, ν is the tuning parameter, $df_{\nu }$ is the number of non-zero coefficients in θ implying the “degrees of freedom” of the estimated EEs, and $B_{n}$ is the scaling factor that diverges to infinity at a slow rate as s→∞. Similarly to [8], when *s* is fixed, we simply take $B_{n}=1$; otherwise, we set $B_{n}=\max\left(\log\log s,1\right)$. By the Bayesian information criterion, it can be seen that the continuously increasing $B_{n}$ is used to offset the effect of the continuously increasing dimension *s* on parameter estimation.

## 3. Asymptotic Properties

### 3.1. Asymptotic Properties for Correctly Specified EEs

This section focuses on the consistent and oracle properties of parameter estimator ${\hat{\theta }}^{PET}$, and on testing the contrast hypothesis. To this end, we need the following assumptions.

**Assumption 1.** *(i) The probability density function f(x,y) is bounded for any x∈X and y∈Y, where X and Y are the supports of x and y, respectively. (ii) The second derivatives of f(x,y) are continuous and bounded.*

**Assumption 2.** *(i) The propensity score function π(u;γ) satisfies $\pi \left(u;\gamma \right)>c_{0}$ for any u∈U, where U is the support of u, and $c_{0}>0$ is a constant. (ii) The second derivatives of π(u;γ) are continuous and bounded.*

**Assumption** **3.** *Parameter vector $\theta ={\left(\beta^{⊤},\gamma^{⊤}\right)}^{⊤}$ is recognizable, which means that there exists a unique solution, $\theta_{0}={\left(\beta_{0}^{⊤},\gamma_{0}^{⊤}\right)}^{⊤}\in \Theta$ to $E[G\left(D_{i};\theta_{0}\right)]=0$ for $D_{i}\in \left\{X,Y,U\right\}$, i=1,…,n.*

**Assumption** **4.** *Let $D_{n\beta }=\{\beta :\|\beta -\beta_{0}\left\|\leq C\sqrt{r/n}\right\}$, $D_{n\gamma }=\{\gamma :\|\gamma -\gamma_{0}\left\|\leq C\sqrt{m/n}\right\}$, and $D_{n\theta }=\{\theta :\|\theta -\theta_{0}\left\|\leq C\sqrt{t/n}\right\}$, where C>0 is a proper constant. There are three constants, $C_{1}^{g}>0$, $K_{1}^{g}>0$, and $K_{2}^{g}>0$, and two functions, $K_{1}^{g}\left({\tilde{D}}_{i}\right)$ and $K_{2}^{g}\left({\tilde{D}}_{i}\right)$, satisfying $E{\left[K_{1}^{g}\left({\tilde{D}}_{i}\right)\right]}^{2}\leq K_{1}^{g}$ and $E{\left[K_{2}^{g}\left({\tilde{D}}_{i}\right)\right]}^{2}\leq K_{2}^{g}$, such that for $\beta \in D_{n\beta }$, (i) $|E\left[n^{-1}\sum_{i=1}^{n}g^{⊤}\left({\tilde{D}}_{i};\beta \right)g\left({\tilde{D}}_{i};\beta \right)\right]|\leq C_{1}^{g}$, with probability tending to one; (ii) $\sup_{j}E{\left[g_{j}\left({\tilde{D}}_{i};\beta \right)\right]}^{2}$$\leq C_{1}^{g}$ and $\sup_{j,l}E{\left[g_{j}\left({\tilde{D}}_{i};\beta \right)g_{l}\left({\tilde{D}}_{i};\beta \right)\right]}^{2}\leq C_{1}^{g}$; (iii) $\sup_{j,l,\beta }\left|\partial g_{j}\left({\tilde{D}}_{i};\beta \right)/\partial \beta_{l}\right|\leq K_{1}^{g}\left({\tilde{D}}_{i}\right)$ and $\sup_{j,l_{1},l_{2},\beta }\left|\partial^{2}g_{j}\left({\tilde{D}}_{i};\beta \right)/\partial \beta_{l_{1}}\partial \beta_{l_{2}}\right|\leq K_{2}^{g}\left({\tilde{D}}_{i}\right)$, where ${\tilde{D}}_{i}=\left\{X_{i},Y_{i}\right\}$.*

**Assumption** **5.** *$t=o(n^{1/2-1/\iota })$ for some ι>2 and t=O(s).*

Assumptions 1 and 2 are the widely used conditions in the missing data literature. Assumption 1 implies that the probability density functions f(u) and f(z) are bounded for any u∈U and z∈Z, respectively, where Z is the support of *z*. Assumption 3 ensures the existence and consistency of the solution that can be obtained with (7). Assumption 4 is a common condition for sample moments. Also, it follows from Assumptions 1(i) and 2 and (3) that the EEs $\varphi (U_{i};\gamma )$ and $g^{ipw}\left(D_{i};\theta \right)$ satisfy Assumption 4 for $\gamma \in D_{n\gamma }$ and $\theta \in D_{n\theta }$. There are constants $C_{1}^{G}>0$, $K_{1}^{G}>0$, $K_{2}^{G}>0$ and functions $K_{1}^{G}\left(D_{i}\right)$ and $K_{2}^{G}\left(D_{i}\right)$ satisfying $E{\left[K_{1}^{G}\left(D_{i}\right)\right]}^{2}\leq K_{1}^{G}$ and $E{\left[K_{2}^{G}\left(D_{i}\right)\right]}^{2}\leq K_{2}^{G}$, such that, for $\theta \in D_{n\theta }$, (i) $|E\left[n^{-1}\sum_{i=1}^{n}G^{⊤}\left(D_{i};\theta \right)G\left(D_{i};\theta \right)\right]|\leq C_{1}^{G}$ with probability tending to one; (ii) $\sup_{j}E{\left[G_{j}\left(D_{i};\theta \right)\right]}^{2}\leq C_{1}^{G}$ and $\sup_{j,l}E{\left[G_{j}\left(D_{i};\theta \right)G_{l}\left(D_{i};\theta \right)\right]}^{2}\leq C_{1}^{G}$; (iii) $\sup_{j,l,\theta }\left|\partial G_{j}\left(D_{i};\theta \right)/\partial \theta_{l}\right|\leq K_{1}^{G}\left(D_{i}\right)$ and $\sup_{j,l_{1},l_{2},\theta }\left|\partial^{2}G_{j}\left(D_{i};\theta \right)/\partial \theta_{l_{1}}\partial \theta_{l_{2}}\right|\leq K_{2}^{G}\left(D_{i}\right)$, which is used to ensure the boundedness of EEs $G(D_{i};\theta )$ and their derivatives [8]. Here, we assume that *t* and *s* are allowed to be divergent when n→∞. But Assumption 5 imposes certain limitations on the divergence rate of *t* and *s*.

**Lemma** **1.** *Under Assumptions 1–5, we have*

$$\begin{array}{c} l_{pn}\left(\theta \right)=-\frac{1}{2}{\left(\theta -\dot{\theta }\right)}^{⊤}V\left(\theta -\dot{\theta }\right)+o_{p}\left(t/n\right), \end{array}$$

*where $V=V_{0}+M\Sigma^{-1}M^{⊤}$, $\dot{\theta }=V^{-1}\left(V_{0}\theta_{0}+M\Sigma^{-1}M^{⊤}{\hat{\theta }}^{\mathrm{ET}}\right)$, ${\hat{\theta }}^{\mathrm{ET}}$ is the ET likelihood estimator of θ, and $V_{0}$ is a diagonal matrix with the jth diagonal element being $V_{0}^{jj}$ for j=1,…,p+q.*

Similarly to [8], the *j*th non-zero diagonal component $V_{0}^{jj}$ can be evaluated with the following quadratic approximation of $p_{\nu }\left(\right|\theta_{j}\left|\right)$ at $\theta_{0j}$: $p_{\nu }\left(\right|\theta_{j}\left|\right)=p_{\nu }\left(\right|\theta_{0j}\left|\right)+{\dot{p}}_{\nu }\left(\right|\theta_{j}^{\xi }\left|\right)\theta_{j}^{\xi }\left(\theta_{j}-\theta_{0j}\right)/|\theta_{j}^{\xi }|\approx p_{\nu }\left(\right|\theta_{0j}\left|\right)+{\dot{p}}_{\nu }\left(\right|\theta_{j}^{\xi }\left|\right)\left(\theta_{j}^{2}-\theta_{0j}^{2}\right)/\left(2\right|\theta_{j}^{\xi }\left|\right)$ for $\theta_{j}\in \{\theta_{j}:|\theta_{j}-\theta_{0j}\left|\leq C\sqrt{t/n}\right\}$, which yields $V_{0}^{jj}={\dot{p}}_{\nu }\left(\right|\theta_{j}^{\xi }\left|)/\right|\theta_{j}^{\xi }|$, where $\theta_{j}^{\xi }$ is a point lying in the line between $\theta_{j}$ and $\theta_{0j}$. Lemma 1 provides a quadratic form of the penalized ET likelihood ratio function, which is approximately an ellipsoid, with $\dot{\theta }$ being the center. Lemma 1 can be used to obtain the consistency and asymptotic properties of the parameter estimator for θ. To obtain the oracle property of the parameter estimator for θ, we also need the following assumptions:

**Assumption** **6.** *For any ϵ>0, $\inf_{\beta \in \Theta_{\beta }^{W}}||E\left[g\left({\tilde{D}}_{i};\beta \right)\right]\left|\right|\geq \psi_{1}\left(d_{\beta },r\right)\psi_{2}\left(\epsilon \right)>0$ and $\inf_{\gamma \in \Theta_{\gamma }^{W}}||E\left[\varphi \left(U_{i};\gamma \right)\right]\left|\right|\geq \psi_{1}\left(d_{\gamma },m\right)\psi_{2}\left(\epsilon \right)>0$, where $\Theta_{\beta }^{W}=\{\beta \in \Theta_{\beta }:||\beta -\beta_{0}\left||\geq \epsilon \right\}$, $\Theta_{\gamma }^{W}=\{\gamma \in \Theta_{\gamma }:||\gamma -\gamma_{0}\left||\geq \epsilon \right\}$, $\Theta_{\beta }$ is the projection of θ-parameter space *Θ* onto β, $\Theta_{\gamma }$ is the projection of θ-parameter space *Θ* onto γ, $\psi_{2}\left(\cdot \right)$ is some proper positive function, and the positive function $\psi_{1}\left(\cdot ,\cdot \right)$ satisfies $\lim\inf_{d_{\beta },r\to \infty }\psi_{1}\left(d_{\beta },r\right)>0$.*

**Assumption** **7.** *Let $\Sigma_{g}\left(\beta \right)=E\left[\left\{g_{i}\left(\beta \right)-g\left(\beta \right)\right\}{\left\{g_{i}\left(\beta \right)-g\left(\beta \right)\right\}}^{⊤}\right]$. There are constants $C_{2}^{g}$ and $C_{3}^{g}$, such that $0<C_{2}^{g}\leq {\mathrm{EV}}_{1}\left\{\Sigma_{g}\left(\beta \right)\right\}\leq \cdots \leq {\mathrm{EV}}_{t}\left\{\Sigma_{g}\left(\beta \right)\right\}\leq C_{3}^{g}<\infty$ for $\forall \beta \in D_{n\beta }$, where $g_{i}\left(\beta \right)=g\left({\tilde{D}}_{i};\beta \right)$, $g\left(\beta \right)=E[g\left({\tilde{D}}_{i};\beta \right)]$, ${\mathrm{EV}}_{j}\left(A\right)$ represents the jth eigenvalue of matrix A.*

**Assumption** **8.** *There is a positive function η(ν)>0, such that η(ν)=O(ν). For $\theta_{0}\in \Theta$, $\max_{j}\left|\theta_{0j}\right|<\infty$, and η(ν) satisfies $\eta \left(\nu \right)/\min_{j\in A}\left|\theta_{0j}\right|\to 0$, where $\theta_{0j}$ represents the jth component of the true value $\theta_{0}$ for parameter vector θ.*

**Assumption** **9.** *The penalty function $p_{\nu }\left(\cdot \right)$ satisfies $\max_{j}{\dot{p}}_{\nu }\left(\right|\theta_{0j}\left|\right)=o\left\{{\left(ns\right)}^{-1/2}\right\}$ and $\max_{j}{\ddot{p}}_{\nu }\left(\right|\theta_{0j}\left|\right)=o\left(s^{-1/2}\right)$, and $\max_{j}{\dot{p}}_{\nu }\left(\right|\theta_{j}\left|\right)/\eta \left(\nu \right)\to 0$ and $s/[\eta \left(\nu \right)\min_{j}{\dot{p}}_{\nu }\left(\right|\theta_{j}\left|)\right]\to 0$ for $\theta \in D_{n\theta }$ as n→∞, where ${\dot{p}}_{\nu }\left(\cdot \right)$ and ${\ddot{p}}_{\nu }\left(\cdot \right)$ are the first- and second-order derivatives of $p_{\nu }\left(\cdot \right)$, respectively.*

Assumption 6 is the identification condition for the parameter space, which grows to infinity. Assumption 7 ensures that $\Sigma_{g}\left(\beta \right)$ is a strictly positive definite matrix. It follows from Assumptions 1(i) and 2(i) and (3) that $\Sigma_{\varphi }\left(\gamma \right)=E\left[\left\{\varphi_{i}\left(\gamma \right)-\varphi \left(\gamma \right)\right\}{\left\{\varphi_{i}\left(\gamma \right)-\varphi \left(\gamma \right)\right\}}^{⊤}\right]$ and $\Sigma_{g^{ipw}}\left(\theta \right)=E\left[\left\{g_{i}^{ipw}\left(\theta \right)-g^{ipw}\left(\theta \right)\right\}{\left\{g_{i}^{ipw}\left(\theta \right)-g^{ipw}\left(\theta \right)\right\}}^{⊤}\right]$ are also strictly positive definite matrices, where $\varphi_{i}\left(\gamma \right)=\varphi \left(U_{i};\gamma \right)$, $\varphi \left(\gamma \right)=E[\varphi \left(U_{i};\gamma \right)]$, $g_{i}^{ipw}\left(\theta \right)=g^{ipw}\left(D_{i};\theta \right)$ and $g^{ipw}\left(\theta \right)=E\left[g^{ipw}\left(D_{i};\theta \right)\right]$. The tuning parameter ν associated with the penalty function controls the variable selection process to some extent. Without loss of generality, we assume that there exists a positive function η(ν) related to ν, such that $\hat{A}=\left\{j:\right|{\hat{\theta }}_{j}^{\mathrm{PET}}\left|>\eta \left(\nu \right)\right\}$, where ${\hat{\theta }}_{j}^{\mathrm{PET}}$ is the *j*th component of the penalized ET estimator ${\hat{\theta }}^{\mathrm{PET}}$. In addition, if parameter vector θ quickly converges to zero, the estimators of parameters will be subject to certain disturbances, which results in the relatively large biases and variances of parameter estimators. Therefore, Assumption 8 limits the convergence rate of parameter vector θ. Assumption 9 has been adopted in the regularization literature. The widely used penalty functions, such as the SCAD penalty function, satisfy Assumption 9.

**Theorem** **1.** *Let ${\overset{ˇ}{\theta }}^{\mathrm{PET}}={\left({\overset{ˇ}{\theta }}_{1}^{\mathrm{PET}⊤},{\overset{ˇ}{\theta }}_{2}^{\mathrm{PET}⊤}\right)}^{⊤}$ be the penalized ET likelihood estimator of θ for a correctly specified model. Under Assumptions 1–9, we have*

*(i)*  *$||{\overset{ˇ}{\theta }}_{1}^{\mathrm{PET}}-\theta_{10}||\overset{P}{⟶}0$ and ${\overset{ˇ}{\theta }}_{2}^{\mathrm{PET}}\overset{P}{⟶}0$ as n→∞, where the notation $\overset{P}{⟶}$ represents the convergence in probability.*
*(ii)*  *$\sqrt{n}A_{n}\Gamma_{1}^{-1/2}\left({\overset{ˇ}{\theta }}^{\mathrm{PET}}-\theta_{0}\right)\overset{L}{⟶}N\left(0,V\right)$, where $A_{n}\in R^{s\times t}$ satisfies $A_{n}A_{n}^{⊤}\to V$ as n→∞, V is a s×s nonnegative symmetric matrix, and $\overset{L}{⟶}$ denotes convergence in distribution.*

When the model correctly specified, Theorem 1 shows that the penalized ET likelihood estimator ${\overset{ˇ}{\theta }}^{\mathrm{PET}}$ of θ possesses the consistency property for non-zero components of $\theta_{0}$, and zero components of $\theta_{0}$ are estimated as zero, with probability tending to one by the penalized ET likelihood method. Also, Theorem 1 shows that the distribution of the penalized ET likelihood estimator ${\overset{ˇ}{\theta }}^{\mathrm{PET}}$ asymptotically converges to the normal distribution. In particular, it follows from ${\overset{ˇ}{\theta }}^{\mathrm{PET}}=\left({\overset{ˇ}{\beta }}^{\mathrm{PET}⊤},{\overset{ˇ}{\gamma }}^{\mathrm{PET}⊤}\right)$ that $\sqrt{n}A_{n,\beta }\Gamma_{1,\beta }^{-1/2}\left({\overset{ˇ}{\beta }}^{\mathrm{PET}}-\beta_{0}\right)\overset{L}{⟶}N\left(0,V_{\beta }\right)$ and $\sqrt{n}A_{n,\gamma }\Gamma_{1,\gamma }^{-1/2}\left({\overset{ˇ}{\gamma }}^{\mathrm{PET}}-\gamma_{0}\right)\overset{L}{⟶}N\left(0,V_{\gamma }\right)$, where $A_{n,\beta }$, $\Gamma_{1,\beta }$, and $V_{\beta }$ are the projections of matrices $A_{n}$, $\Gamma_{1}$, and V onto the β space, respectively, and $A_{n,\gamma }$, $\Gamma_{1,\gamma }$, and $V_{\gamma }$ are the projections of matrices $A_{n}$, $\Gamma_{1}$, and V onto the γ space, respectively.

We consider the following null and alternative hypotheses for testing the contrast of parameters θ:

$$\begin{array}{c} H_{0}:C_{n}\theta =0\;\mathrm{versus}\;H_{1}:C_{n}\theta \neq 0, \end{array}$$

where $C_{n}\in R^{e\times s}$ satisfies $C_{n}C_{n}^{⊤}=I_{e}$ for some fixed integer *e* and $I_{e}$ is the e×e identity matrix. The aforementioned hypothesis includes some special cases. For example, $H_{0j}$: $\theta_{j}=0$ for j=1,…,s, or $H_{0}$: $\theta_{j}-\theta_{k}=0$ for j≠k∈{1,…,s}, and so on. The penalized ET likelihood ratio statistic for testing the null hypothesis $H_{0}:C_{n}\theta =0$ is given as

(9) $$\begin{array}{c} \tilde{l}\left(C_{n}\right)=2n\left\{l_{pn}\left({\hat{\theta }}^{\mathrm{PETO}}\right)-\max_{\theta ,C_{n}\theta =0}l_{pn}\left(\theta \right)\right\}, \end{array}$$

where ${\hat{\theta }}^{\mathrm{PETO}}=\arg\max_{\theta \in \Theta }\inf_{\lambda \in {\hat{\Lambda }}_{n}\left(\theta \right)}l_{pn}\left(\theta \right)$. Similarly to the empirical likelihood literature, we can show that $\tilde{l}\left(C_{n}\right)$ converges to $\chi^{2}$-distribution as n→∞ under some regularity conditions.

### 3.2. Asymptotic Properties for Misspecified EEs

Section 3.1 discusses the asymptotic properties of estimators for parameters θ when EEs are correctly specified. However, in practical applications, it is rather difficult to correctly specify EEs. That is, misspecified EEs are usually encountered in some applications. Thus, the aforementioned asymptotic properties do not hold for misspecified EEs. To address this issue, here we investigate the asymptotic properties of estimators for parameters θ obtained with misspecified EEs. To this end, we need the following assumptions.

**Assumption 10.** (i) The EEs $g(X_{i};\beta )$ and penalty function $p_{\nu }\left(|\theta |\right)$ are continuous. (ii) The second-order derivatives of $g(X_{i};\beta )$ and $\pi (U_{i};\gamma )$ are continuous with respect to β and γ in the neighbourhoods $N_{\beta }^{*}$ and $N_{\gamma }^{*}$ of $\beta^{*}$ and $\gamma^{*}$, respectively, where $\beta^{*}$ and $\gamma^{*}$ are the “pseudo-truth” values of β and γ, which are interior points of $\Theta_{\beta }$ and $\Theta_{\gamma }$, respectively.

**Assumption** **11.** There are constant $K_{3}^{G}<\infty$ and functions $K_{3}^{G}\left(D_{i}\right)$ satisfying the condition $E{\left\{K_{3}^{G}\left(D_{i}\right)\right\}}^{2}\leq K_{3}^{G}$, such that $\sup_{\theta \in \Theta }\sup_{\lambda \in {\hat{\Lambda }}_{n}\left(\theta \right)}\exp\left\{\lambda^{⊤}G\left(D_{i};\theta \right)\right\}<K_{3}^{G}\left(D_{i}\right)$, where ${\hat{\Lambda }}_{n}\left(\theta \right)$ is a compact set, such that $\lambda^{*}\left(\theta \right)\in \mathrm{int}\left({\hat{\Lambda }}_{n}\left(\theta \right)\right)$.

**Assumption 12.**(i) For all $\theta \in R^{s}$, the conditional expectation $E\left\{G\left(D_{i};\theta \right)|G\left(D_{i};\theta_{0}\right)=G\left(d;\theta_{0}\right)\right\}=g\left(d;\theta_{0}\right)$ exists. (ii) The expectation function $E[\exp\left\{\lambda^{*⊤}\left(\theta \right)G\left(D_{i};\theta \right)\right\}]$ is convex and maximized at the “pseudo-truth” value $\theta^{*}$ of θ, where $\theta^{*}$ *is the interior point of Θ*.

**Assumption** **13.** *There is a function $h(D_{i})$ satisfying*

$$E\left[\underset{\theta \in N_{\theta }^{*}}{\sup}\underset{\lambda \in {\hat{\Lambda }}_{n}\left(\theta \right)}{\sup}\exp\left\{l_{1}\lambda^{⊤}G\left(D_{i};\theta \right)\right\}h^{l_{2}}\left(D_{i}\right)\right]<\infty$$

for $l_{1},l_{2}=0,1,2$, where $N_{\theta }^{*}$ is the neighbourhood of $\theta^{*}$, such that $||G\left(D_{i};\theta \right)\left|\right|\leq h\left(D_{i}\right)$, $\left|\right|\partial_{\theta }G\left(D_{i};\theta \right)\left|\right|\leq h\left(D_{i}\right)$, $\left|\right|\partial_{\theta_{j_{1}}\theta_{j_{2}}}G\left(D_{i};\theta \right)\left|\right|\leq h\left(D_{i}\right)$ for any $\theta \in N_{\theta }^{*}$ and $j_{1},j_{2}=1,\ldots ,s$, where $\partial_{\theta }G\left(D_{i};\theta \right)≐\partial G\left(D_{i};\theta \right)/\partial \theta$ and $\partial_{\theta_{j_{1}}\theta_{j_{2}}}G\left(D_{i};\theta \right)≐\partial^{2}G\left(D_{i};\theta \right)/\partial \theta_{j_{1}}\partial \theta_{j_{2}}$.

**Assumption 14.** *(i) As n→∞ and ν→0, $\lim\inf_{\theta \to 0^{+}}{\dot{p}}_{\nu }\left(\theta \right)/\nu >0$ and ||λ(θ)||=o(ν) for $\lambda \left(\theta \right)\in \mathrm{int}({\hat{\Lambda }}_{n}\left(\theta \right))$. (ii) There is a constant $C_{p}$, such that $\max_{j\in A_{\theta }}{\dot{p}}_{\nu }\left(\right|\theta_{0j}\left|\right)\leq C_{p}\nu$ and $\max_{j\in A_{\theta }}{\ddot{p}}_{\nu }\left(\right|\theta_{0j}\left|\right)\leq C_{p}\nu$.*

Assumptions 10–13 are some regularity conditions on the EEs $g(X_{i},Y_{i};\beta )$, $G(D_{i};\theta )$, and the propensity score functions $\pi (U_{i};\gamma )$ required for the consistency and asymptotic normality of the penalized ET likelihood estimator when EEs are misspecified [8]. Assumptions 11–13 are slightly stronger than Assumptions 1 and 2. In fact, we only need to make corresponding assumptions on the EEs $g(X_{i},Y_{i};\beta )$ and propensity score functions $\pi (U_{i};\gamma )$ based on Assumptions 1 and 2 and Equation (3), and can then obtain Assumptions 11–13. Assumption 14 gives the relationship between the order of Lagrange multipliers of the ET likelihood function and the order of tuning parameter associated with the penalty function, and presents the constraint for the first- and second-order derivatives of the penalty function in terms of the tuning parameter ν, which is used to ensure the sparsity of the penalized ET likelihood estimator in the presence of EE misspecification.

**Theorem** **2.** *Let ${\hat{\theta }}^{\mathrm{PET}}={\left({\hat{\theta }}_{1}^{\mathrm{PET}⊤},{\hat{\theta }}_{2}^{\mathrm{PET}⊤}\right)}^{⊤}$ be the penalized ET likelihood estimator of θ in the presence of EE misspecification. Under Assumptions 10–14, as n→∞, we have*

*(i)*  (Consistency) ${\hat{\theta }}^{\mathrm{PET}}\overset{P}{⟶}\theta^{*}$, where $\overset{P}{⟶}$ denotes convergence in probability;
*(ii)*  *(Oracle property) ${\hat{\theta }}_{2}^{\mathrm{PET}}=0$ with probability tending to one;*
*(iii)*  (Asymptotic normality) $\sqrt{n}\left({\hat{\zeta }}^{\mathrm{PET}}-\zeta^{*}\right)\overset{L}{⟶}N\left(0,\Delta^{-1}\Omega \Delta^{-⊤}\right)$, where $\zeta ={\left(\lambda^{⊤},\theta^{⊤},\tau^{⊤}\right)}^{⊤}$, $\Delta =E\left\{\partial_{\zeta }\tilde{S}\left(\lambda ,\theta ,\tau \right)\right\}{|}_{\zeta =\zeta^{*}}$, $\Omega =E\{\tilde{S}\left(\lambda^{*},\theta^{*},\tau^{*}\right){\tilde{S}}^{⊤}\left(\lambda^{*},\theta^{*},\tau^{*}\right)\}$, τ is the Lagrange multiplier, vector $\tilde{S}\left(\lambda ,\theta ,\tau \right)$ is the first-order derivatives of the penalized log-ET likelihood ratio function for misspecified EEs under constraint $M_{2}\theta =\theta_{2}=0$ in which $M_{2}$ is the projection matrix, and $\overset{L}{⟶}$ denotes convergence in distribution.

Theorem 2 indicates that there is no convergence rate due to the misspecification of EEs; the penalized ET likelihood estimator of θ converges to its pseudo-true value rather than to its true value. Also, Theorem 2 is shown to possess the oracle property under some regularity conditions. The consistency of the estimator holds only when the penalized log-ET likelihood ratio function converges to its population form, satisfying some continuity and boundness assumptions. Moreover, Theorem 3 establishes the asymptotic normality of estimators for the non-zero components of θ.

**Theorem** **3.** *Under Assumptions 10–14, we have $\tilde{l}\left(C_{n}\right)\tilde{M}\overset{L}{⟶}\chi_{e}^{2}$, where $\tilde{M}=K_{1}{\left(S_{1}-S_{2}\right)}^{-1}$ with $K_{1}$, $S_{1}$, and $S_{2}$ defined in Appendix A and $\chi_{e}^{2}$ denotes the chi-squared distribution with e degrees of freedom.*

Theorem 3 extends the results of the growing dimensional unconditional moment models given in [8] to the MAR case. Theorem 3 can be used to test hypotheses and construct the confidence regions of parameters θ. In particular, the 100(1−α)% approximate confidence region for $C_{n}\theta$ based on the constrainedly penalized ET likelihood ratio statistic can be written as

$$CR_{\alpha }=\left\{\varsigma :2n\tilde{M}\left(l_{pn}\left(\hat{\theta }\right)-\max_{\theta ,C_{n}\theta =\varsigma }l_{pn}\left(\theta \right)\right)\leq \chi_{e}^{2}\left(1-\alpha \right)\right\},$$

where $\chi_{e}^{2}\left(1-\alpha \right)$ is the (1−α)-quantile of the chi-squared distribution with *e* degrees of freedom.

## 4. Simulation Studies

In this section, some simulation studies are conducted to investigate the finite sample performance of the proposed methodologies.

*Experiment 1* (Linear Regression Model). The data $\left\{Y_{i}:i=1,\ldots ,n\right\}$ are generated with the following linear regression model: $Y_{i}=\beta_{c}+X_{i}^{⊤}\beta_{x}+\epsilon_{i}$, where $X_{i}={\left(U_{i}^{⊤},Z_{i}^{⊤}\right)}^{⊤}$ is the *p*-dimensional covariate, $\beta_{c}$ is the constant term, $\beta_{x}$ is the *p*-dimensional vector of regression coefficients, the instrumental variables $Z_{i}={\left(z_{i,1},\ldots ,z_{i,p-q}\right)}^{⊤}$ are drawn from the multivariate normal distribution N(0,Σ), i.e., $Z_{i}∼N\left(0,\Sigma \right)$, covariates $U_{i}={\left(u_{i,1},\ldots ,u_{i,q}\right)}^{⊤}$ and $u_{i,j}∼N\left(10z_{i,j},1\right)$ for j=1,…,q (2q=p), and $\epsilon_{i}$’s follow the standard normal distribution N(0,1) for i=1,…,n. The true value $\beta_{0}$ of $\beta ={\left(\beta_{c},\beta_{x}^{⊤}\right)}^{⊤}\in R^{p+1}$ is taken as $\beta_{0}={\left(1.0,0.6,0.5,0.0,\ldots ,0.0\right)}^{⊤}$, which implies that there are two non-zero elements and p−2 zero elements in $\beta_{x}$, corresponding to two active and p−2 inactive covariates. The true value of the (k,l)th component of $\Sigma =(\rho_{kl})$ is taken to be $\rho_{kl}=0.5^{\left|k-l\right|}$ for k,l=1,…,p. Here, we assume that covariates $x_{i}$ are completely observed, while responses $y_{i}$ are subject to missingness. To this end, we regard $\delta_{i}$ as the missing indicator for $Y_{i}$, i.e., $\delta_{i}=0$ if $Y_{i}$ is missing and $\delta_{i}=1$ if $Y_{i}$ is observed, and denote $\pi_{i}\left(U_{i};\gamma \right)=\mathrm{Pr}\left(\delta_{i}=1|U_{i}\right)$. To create missing data for response $y_{i}$, we consider the following propensity score functions:

(M1) $\mathrm{logit}\left\{\pi_{i}\left(U_{i};\gamma \right)\right\}=\gamma_{c}+U_{i}^{⊤}\gamma_{u}$, where $U_{i}={\left(u_{i1},\ldots ,u_{iq}\right)}^{⊤}$ are simulated from the above normal distribution, and $\gamma ={\left(\gamma_{c},\gamma_{u}^{⊤}\right)}^{⊤}={\left(\gamma_{0},\gamma_{1},\ldots ,\gamma_{q}\right)}^{⊤}$, logit(b)=log{b/(1−b)};

(M2) $\pi_{i}\left(U_{i};\gamma \right)=0.5$ if $\gamma_{c}+U_{i}^{⊤}\gamma_{u}\leq a$, and $\pi_{i}\left(U_{i};\gamma \right)=0.5$ if $\gamma_{c}+U_{i}^{⊤}\gamma_{u}>a$;

(M3) $\pi_{i}\left(U_{i};\gamma \right)=\Phi \left(\gamma_{c}+U_{i}^{⊤}\gamma_{u}\right)$, where Φ(·) is the cumulative distribution function of the standard normal distribution.

The true value $\gamma_{0}$ of $\gamma \in R^{q+1}$ is taken as $\gamma_{0}={\left(1.0,0.8,0.5,0.0,\ldots ,0.0\right)}^{⊤}$, which indicates that there are three non-zero components and q−2 zero elements in $\gamma_{0}$ corresponding to two active predictors and q−2 inactive predictors. To illustrate the proposed method, we consider the following unconditional moments: $g\left(D_{i};\beta \right)=Z_{i}\left(Y_{i}-\beta_{c}-Z_{i}^{⊤}\beta_{x}\right)$ and

$$\varphi \left(U_{i},Z_{i};\gamma \right)=\left(\frac{\delta_{i}}{\pi_{i}\left(U_{i};\gamma \right)}-1\right)Z_{i},\;i=1,\ldots ,n$$

with $\pi_{i}\left(U_{i};\gamma \right)$ specified in model (M1), which implies that (M1) is a correctly specified model and (M2) and (M3) are the misspecified models that are used to show the robustness of the proposed methods, and $E\{g\left(D_{i};\beta_{0}\right)\}=0$ and $E\{\varphi \left(U_{i},Z_{i};\theta_{0}\right)\}=0$.

We consider five combinations of (n,p+1,q+1): (n,p,q)=(50,9,5), (100,13,7), (200,17,9), (500,23,12), and (500,101,51), where *p* is the even-integer part of ${\left(5n\right)}^{\frac{2}{5}}$ or $9{\left(n\right)}^{\frac{2}{5}}-8$, and q=p/2. The tuning parameter τ is selected with the BIC, and the SCAD is taken as the penalty function $p_{\nu }\left(\cdot \right)$. For each of the five combinations for (n,p,q), we simulate 1000 datasets, which are used to estimate parameters θ={β,γ} via the proposed method. We calculate the bias, standard deviation (SD), and root mean square error (RMSE) of parameters θ for assessing the accuracy of the proposed method, where bias=$T^{-1}\sum_{t=1}^{T}\theta_{j}^{\left(t\right)}-\theta_{0j}$ (i.e., the difference between the true value and the mean of the estimates based on T=1000 replications), SD=$\sqrt{T^{-1}\sum_{t=1}^{T}{\left(\theta_{j}^{\left(t\right)}-{\bar{\theta }}_{j}\right)}^{2}}$ (i.e., the standard deviation of T=1000 estimates), and $\mathrm{RMSE}=\sqrt{T^{-1}\sum_{t=1}^{T}{\left(\theta_{j}^{\left(t\right)}-\theta_{0j}\right)}^{2}}$ (i.e., the root mean square error of estimates obtained with T=1000 replications and its true value) and ${\bar{\theta }}_{j}=T^{-1}\sum_{t=1}^{T}\theta_{j}^{\left(t\right)}$, in which $\theta_{j}^{\left(t\right)}$ is the penalized ET likelihood estimate of the *j*th component of θ for the *t*th replication for j=1,…,p+q+2. To assess the oracle property of the proposed variable selection method, we evaluate the average number of correctly estimated zero components (denoted as ‘TP’) and the average number of incorrectly estimated zero components (denoted as ‘FP’). Also, to assess the model fitting effect, we calculate the average number of under-fitting (denoted as ‘UF’), correctly-fitting (denoted as ‘CF’), and over-fitting (denoted as ‘OF’) for 1000 repeated simulations, where under-fitting refers to the case that some zero-components are incorrectly estimated as non-zero, over-fitting refers to the case that some non-zero-components are incorrectly estimated to be zero and all zero-components are correctly estimated to be zero, while correctly-fitting refers to the case that all zero-components are correctly estimated to be zero and all non-zero-components are correctly estimated as non-zero. The results for bias, SD, and RMSE values of non-zero parameters in θ and variable selection are given in Table 1. An examination of Table 1 shows that (i) bias values of all the parameter estimates are relatively small and SD and RMSE values of all the parameter estimates are almost identical; (ii) as the sample size *n* increases, the average number of correctly estimated zero components tends towards 100% and the average number of incorrectly estimated zero components tends towards 0, implying that the proposed variable selection method performs well; (iii) as the sample size *n* increases, UF and OF tend towards 0 and CF tends towards 100%; and (iv) although UF, CF, and OF values corresponding to misspecified models (e.g., M2 and M3) are larger than those corresponding to the correctly specified model (e.g., M1), their differences are relatively small, implying that the proposed penalized ET likelihood method is robust to the misspecified model.

*Experiment 2* (logistic model). The data $\left\{Y_{i}:i=1,\ldots ,n\right\}$ are generated from the following logistic model: $Y_{i}∼\mathrm{Bernoulli}\left(p_{i}\right)$ with $\mathrm{logit}\left(p_{i}\right)=\beta_{c}+X_{i}^{⊤}\beta_{x}$, where $X_{i}={\left(U_{i}^{⊤},Z_{i}^{⊤}\right)}^{⊤}$ is the *p*-dimensional covariates, the instrumental variables $Z_{i}∼N_{p}\left(0,I\right)$, where I is a p×p identity matrix, and $\beta ={\left(\beta_{c},\beta_{x}^{⊤}\right)}^{⊤}$. The *q*-dimensional covariates $U_{i}={\left(u_{i,1},\ldots ,u_{i,q}\right)}^{⊤}$ and $u_{i,j}∼N\left(z_{i,j},100\right)$ for j=1,…,q (2q=p). Similarly to experiment 1, we assume that $X_{i}$’s are fully observed, while $Y_{i}$’s are subject to missingness and missing indicators $\delta_{i}$ for responses $Y_{i}$ are simulated from the Bernoulli distribution with the propensity score function $\pi_{i}\left(U_{i};\gamma \right)$ and $\mathrm{logit}\left\{\pi_{i}\left(U_{i};\gamma \right)\right\}=\gamma_{c}+U_{i}^{⊤}\gamma_{u}$, where $\gamma ={\left(\gamma_{c},\gamma_{u}^{⊤}\right)}^{⊤}$. Here, we consider the same true value of {θ,γ} as those given in experiment 1, four combinations of triples (n,p+1,q+1): (n,p+1,q+1)=(50,9,5), (100,13,7), (200,17,9), and (500,101,51), where *p* is the even-integer part of ${\left(5n\right)}^{\frac{2}{5}}$ or $9n^{\frac{2}{5}}-8$ and q=p/2. As an illustration of the proposed method, we consider the following unconditional moment constraints:

$$g\left(D_{i};\beta \right)={\left(1,Z_{i}\right)}^{⊤}\left\{Y_{i}-\frac{\exp(\beta_{c}+Z_{i}^{⊤}\beta_{x})}{1+\exp(\beta_{c}+Z_{i}^{⊤}\beta_{x})}\right\},\;\;\;i=1,\ldots ,n.$$

The missing mechanism model for fitting data is given in Equation (1). We select the tuning parameter τ with the BIC criterion and take the SCAD as the penalty function. For simplicity, let $\omega^{1}=n^{-1}\sum_{i=1}^{n}{\left(1,Z_{i}\right)}^{-⊤}\log\left({\tilde{p}}_{i}/\left(1-{\tilde{p}}_{i}\right)\right)$, $\omega^{2}=n^{-1}\sum_{i=1}^{n}{\left(1,U_{i}\right)}^{-⊤}\log\left({\tilde{\pi }}_{i}/\left(1-{\tilde{\pi }}_{i}\right)\right)$ and $\omega ={\left(\omega^{1⊤},\omega^{2⊤}\right)}^{⊤}$ is a *s*-dimensional vector, where ${\tilde{p}}_{i}=n^{-1}\sum_{i=1}^{n}y_{i}$, ${\tilde{\pi }}_{i}=n^{-1}\sum_{i=1}^{n}\delta_{i}$, $\omega_{j}$ is the *j*th component of ω, I(A) is an indicator function of the event *A*, and ${\left\{B\right\}}_{+}=\max\left(0,B\right)$. For comparison, we consider the hard-threshold (HT) estimator ${\hat{\theta }}_{j}^{\mathrm{HT}}=\omega_{j}I(|\omega_{j}\left|<\varsigma_{1}\right)$, the soft-threshold (ST) estimator ${\hat{\theta }}_{j}^{\mathrm{ST}}=\mathrm{sign}\left(\omega_{j}\right)\left\{\right|\omega_{j}{\left|-\varsigma_{2}\right\}}_{+}$, and the penalized least squares estimator with the SCAD penalty function (denoted as ‘PLS’). In implementing the proposed method (denoted as ‘PETL’), we evaluate the tuning parameters $\left\{\varsigma_{1},\varsigma_{2}\right\}$ with the five-fold cross-validation method. The corresponding results of non-zero parameters in β and γ and variable selection for 1000 replications are given in Table 2.

An examination of Table 2 shows that (i) the proposed method and the competing methods have relatively small Bias values and negligible differences between SD and RMSE values, regardless of the sample size and the dimensionality, which implies that the compared four methods can well estimate non-zero parameters; (ii) the proposed method has larger TP and CF values and smaller FP and UF values than the compared three methods, regardless of the sample size and the dimensionality, which implies that the proposed method behaves better than the compared three methods; (iii) as the sample size increases, the TP and CF values of the proposed method approach p−3, while its FP, UF, and OF values approach 0 and its CF values are close to one; (iv) the HT and ST methods for variable selection perform poorly due to the lack of the penalty function; and (v) when the sample size is small, the PLS method for variable selection behaves unsatisfactorily, even if the SCAD penalty function is used. In a word, the proposed method outperforms the other three methods, regardless of parameter estimation and variable selection.

*Experiment 3* (structural equation model). To investigate the performance of the proposed method, we consider a more complicated model (e.g., structural equation model), which is specified by

$$Y_{i}=BX_{i}+\epsilon_{i},\;\;\;X_{i}=CX_{i}+\varepsilon_{i},\;\;i=1,\ldots ,n$$

where $Y_{i}$ is an *e*-dimensional vector of manifest variables, $X_{i}={\left(U_{i}^{⊤},Z_{i}^{⊤}\right)}^{⊤}$ is a *f*-dimensional vector of latent variables, where $U_{i}=\left(u_{i,1},\ldots ,u_{i,h}\right)$ is an *h*-dimensional vector, $Z_{i}=\left(z_{i,1},\ldots ,z_{i,f-h}\right)$ is a f−h-dimensional instrumental variables and f=2h, *B* is a e×f factor loading matrix, *C* is a f×f coefficient matrix used to identify the dependence structure between latent variables, and $\epsilon_{i}$ and $\varepsilon_{i}$ are measurement errors. It is assumed that $\epsilon_{i}$’s follow the multivariate normal distribution with zero mean and covariance $\Sigma_{\epsilon }=\mathrm{diag}\left(\alpha_{1},\ldots ,\alpha_{e}\right)$, i.e., $\epsilon_{i}∼N\left(0,\Sigma_{\epsilon }\right)$, $\varepsilon_{i}={\left(\varepsilon_{u,i}^{⊤},\varepsilon_{z,i}^{⊤}\right)}^{⊤}$, where $\varepsilon_{u,i}$ is an *h*-dimensional zero vector and $\varepsilon_{z,i}$’s follow the multivariate normal distribution $N(0,\Sigma_{\varepsilon })$, where $\Sigma_{\varepsilon }=\mathrm{diag}\left(\zeta_{1},\ldots ,\zeta_{f-h}\right)$ and e=2f. The dataset ${\left\{Y_{i}\right\}}_{i=1}^{n}$ is generated from the structural equation model introduced above, with the following specification of *B* and *C*:

$$B=\left(\begin{array}{ccc} \beta_{1,1} & \cdots & \beta_{1,f}\\ \vdots & \ddots & \vdots \\ \beta_{e,1} & \cdots & \beta_{e,f} \end{array}\right),\;\;\;\;C=\left(\begin{array}{cc} 0 & C_{u}\\ 0 & C_{z} \end{array}\right)$$

where

$$C_{u}=\left(\begin{array}{ccccc} 1 & 0.5 & \cdots & 0.0 & 0.0\\ -0.5 & 1 & \cdots & 0.0 & 0.0\\ \vdots & \vdots & \ddots & \vdots & \vdots \\ 0.0 & 0.0 & \cdots & 1 & 0.5\\ 0.0 & 0.0 & \cdots & -0.5 & 1 \end{array}\right),\;\;\;\;C_{z}=\left(\begin{array}{ccccc} 1 & 0.8 & \cdots & 0.0 & 0.0\\ -0.8 & 1 & \cdots & 0.0 & 0.0\\ \vdots & \vdots & \ddots & \vdots & \vdots \\ 0.0 & 0.0 & \cdots & 1 & 0.8\\ 0.0 & 0.0 & \cdots & -0.8 & 1 \end{array}\right)$$

are the h×(f−h) and (f−h)×(f−h) matrices, respectively. Here, we take the true values of $\zeta_{m}$ and $\alpha_{k}$ as 0.8 for m=1,…,f−h and k=1,…,e, and set the true value of $\beta_{j_{1},j_{2}}$ to be 0.6 for $|j_{1}-j_{2}|=1$ and 0.0 otherwise, which indicates that the number of non-zero parameters is 2(f−1). Because $\beta_{k,l}$ measures the dependence of manifest variables $Y_{i,k}$ on latent variable $X_{i,l}$, we only regard $\beta_{k,l}$ as a parameter of interest, which indicates that $p=ef=2f^{2}$. To create the missing values of components of $Y_{i}$ for i=1,…,n, we consider the missingness data mechanism model given in Equation (1). The true value of the *q*-dimensional parameter vector $\gamma ={\left(\gamma_{c},\gamma_{u}^{⊤}\right)}^{⊤}$ in Equation (1) is taken as γ=(0.8,−0.8,0.0,…,0.0) and q=h+1. As an illustration of the proposed method, we consider the following unconditional moment constraints:

(10) $$\begin{array}{c} g\left(Y_{i};\beta \right)=\mathrm{vech}\left\{Y_{i}Y_{i}^{⊤}-B{\left(I-C\right)}^{-1}\Sigma_{\epsilon }{\left(I-C\right)}^{-⊤}B^{⊤}+\Sigma_{\varepsilon }\right\}, \end{array}$$

which satisfy $E[g\left(Y_{i};\beta_{0}\right)]=0$ for i=1,…,n, where $\beta_{0}$ is the true value of β, and vech(H) represents the half-vectorization of matrix *H*. In this case, the number of unconditional moment constraints is t=f(2f+1)+h, which shows that $g(Y_{i};\beta )$’s are over-identification conditions (i.e., t>s). Under the settings specified above, we consider the following three combinations of the number of parameters of interest *p*, the number of parameters *q* in missingness data mechanism model (1), and the sample size *n*: (n,p,q)=(117,8,2),(241,32,3),(602,72,4), where *n* is taken as the integer of ${\left(\left(p+80\right)/18\right)}^{3}$. For comparison, we also consider the following two competing methods: generalized method of moments (GMM) and penalized empirical likelihood (PEL). For each of combinations of (n,p,q), we generate 1000 datasets. For each of the simulated 1000 datasets, we utilize the proposed method and the considered two competing methods to estimate parameters and select variable selection. In implementing the proposed method, we use BIC criterion to select the tuning parameters τ. Bias, standard deviation (SD), and root mean square error (RMSE) values of the non-zero component of *C* for 1000 replications are reported in Table 3. Also, the TP, FP, UF, CF, and OF values for identifying non-zero components of *C* are given in Table 4.

An examination of Table 3 and Table 4 shows that (i) SD values of the parameter estimates are almost the same as their corresponding RMSE values for the considered three methods, regardless of the sample sizes, which implies that the considered three methods can estimate parameters well; (ii) the RMSE value decreases as the sample size increases for the considered three methods, which indicates that increasing the sample size can improve the performance of the considered three estimation methods; (iii) the proposed penalized ET likelihood estimation behaves better than the other two competing methods, in that the total RMSE value of the former is smaller than that of the latter, while the GMM estimation has the same performance as the PEL estimation, in that they have the same total RMSE value, indicating that the GMM and PEL methods have the same asymptotic properties [24]; (iv) and the PETL method behaves better than the other two methods in identifying non-zero components of *C*, in that the former has smaller FP, UF, and OF values and a larger CF value than the latter, and the TP value of the former is closer to the number of zero components in *C* (i.e., ${\left(f-1\right)}^{2}+1$) than that of the latter, regardless of the sample sizes.

## 5. Real Examples

As an illustration of the proposed method, here we consider thyroid data taken from the First People’s Hospital of Yunnan Province. In the data, thyroid nodule refers to any growth of thyroid cells that form a lump within the thyroid. Most thyroid nodules do not cause any symptoms. Mostly, a nodule cannot cause pain, difficulty swallowing or breathing, hoarseness, or symptoms of hyperthyroidism; these are usually defined as benign nodules. Here, we explore the relationship between malignant thyroid nodules and variables obtained with ultrasound (US) instruments and clinical characteristics, such as nodular echogenecity ($x_{1}$), thyroid background echogenecity ($x_{2}$), anterior cervical muscles echogenecity ($x_{3}$), echo ratio between nodular and anterior cervical muscles ($x_{4}$), nodule size ($x_{5}$), longitudinal diameter ($x_{6}$), horizontal diameter ($x_{7}$), aspect ratio ($x_{8}$), age ($x_{9}$), sex (1 = male, 0 = female; $x_{10}$), number of nodules (1 = more, 0 = less; $x_{11}$), shape (1 = irregularities, 0 = rules; $x_{12}$), margins (1 = unclear, 0 = clear; $x_{13}$), margins (1 = angled, 0 = non-angled; $x_{14}$), echogenecity (1 = uniform, 0 = uneven; $x_{15}$), internal structural solidity (1 = yes, 0 = no; $x_{16}$), internal structure cystic (1 = yes, 0 = no; $x_{17}$), internal structure cystic solid mixture (1 = yes, 0 = no; $x_{18}$), sound halo (1 = yes, 0 = no; $x_{19}$), rear attenuation (1 = yes, 0 = no; $x_{20}$), lateral acoustic shadow (1 = yes, 0 = no; $x_{21}$), bloodstream (1 = yes, 0 = no; $x_{22}$), blood supply branch (1 = yes, 0 = no; $x_{23}$), blood supply strip (1 = yes, 0 = no; $x_{24}$), blood supply punctate (1 = yes, 0 = no; $x_{25}$), and calcification (1 = yes, 0 = no; $x_{26}$). Thus, we take the aforementioned 26 variables as covariates, denoted as $x\;=\;{\left(x_{1},\ldots ,x_{26}\right)}^{⊤}$, and regard the indicator of malignant thyroid nodules (1 = malignant, 0 = benign) as response variable *y*, i.e., y=1 if patient has the malignant thyroid nodules, and y=0 otherwise. In this study, the sample size is n=2430 and covariates in *x* are fully observed, while roughly 40% of observations of response variable *y* are subject to missingness. Here, we assume that the missingness data mechanism for *y* is missing at random, in that *y* can be unobserved due to the presence of some covariates rather than judgement of benign or malignant thyroid nodules. Under the above assumption, we consider the following logistic regression model:

$$\mathrm{logit}\left\{\mathrm{Pr}\left(y=1|x\right)\right\}=\beta_{0}+x_{1}\beta_{1}+\cdots +x_{26}\beta_{26},$$

which leads to the following unconditional moment constraints:

$$g\left(x_{i},y_{i};\theta \right)={\left(1,x_{i}\right)}^{⊤}\left\{y_{i}-\frac{\exp(\beta_{0}+x_{i1}\beta_{1}+\cdots +x_{i,26}\beta_{26})}{1+\exp(\beta_{0}+x_{i1}\beta_{1}+\cdots +x_{i,26}\beta_{26})}\right\},$$

which satisfy $E\{g\left(x_{i},y_{i};\theta \right)\}=0$, where $\theta ={\left(\beta_{0},\beta_{1},\ldots ,\beta_{26}\right)}^{⊤}$ and $x_{i}={\left(x_{i,1},\ldots ,x_{i,26}\right)}^{⊤}$ for i=1,…,n. Let $U_{i}=\left(x_{i,1},x_{i,4},x_{i,8},x_{i,14},x_{i,15},x_{i,18},x_{i,25}\right)$, and other components of $x_{i}$ are taken as instrumental variables (denoted as $U_{i}$), in that the components in $U_{i}$ can be indirectly determined by the components in $Z_{i}$; for example, when the blood supply is neither branching nor strip-shaped, it can be determined as point-shaped. For missing value of $y_{i}$’s, we consider a logistic regression model for propensity score function $\pi_{i}\left(U_{i};\gamma \right)$, i.e., $\mathrm{logit}\{\pi_{i}\left(U_{i};\gamma \right)\}=\gamma_{c}+U_{i}^{⊤}\gamma_{u}$ with $\gamma ={\left(\gamma_{c},\gamma_{1},\ldots ,\gamma_{q}\right)}^{⊤}$ and q=7. To apply the preceding proposed method to the considered dataset, we consider the following unconditional moments for estimating unknown parameters in γ:

$$\varphi \left(U_{i},Z_{i};\gamma \right)=\left(\frac{\delta_{i}}{\pi_{i}\left(U_{i};\gamma \right)}-1\right)Z_{i},\;\;\;\;i=1,\ldots ,n.$$

In utilizing the proposed method, we take the tuning parameter τ with the BIC criterion and consider three penalty functions, Lasso, Adaptive Lasso (represented by ALasso), and SCAD for comparison. We calculate the PETL estimates of unknown parameters and their corresponding 95% confidence intervals.

An examination of Table 5 shows that the three penalty functions consistently identify $\beta_{2},\beta_{4},\beta_{5},\beta_{6},\beta_{7},\beta_{10},\beta_{16},\beta_{17},\beta_{18},\beta_{19},\beta_{21}$ as zero, in that their corresponding 95% CIs contain zero, and consistently detect $\beta_{11},\beta_{12},\beta_{13},\beta_{14},\beta_{15},\beta_{20},\beta_{22},\beta_{26}$ as non-zero, in that their corresponding 95% CIs do not contain zero (i.e., number of nodules, shape, margins, echogenecity, rear attenuation, bloodstream, and calcification are positively associated with malignant thyroid nodules), while $\beta_{3}$ and $\beta_{9}$ are identified as no effect on malignant thyroid nodules by Lasso and ALasso penalty functions but there ia a negative effect on malignant thyroid nodules only by SCAD. Also, $\beta_{23}$, $\beta_{24}$, and $\beta_{25}$ are detected as on effect on malignant thyroid nodules only by Lasso. The PETL estimates of non-zero parameters obtained with three penalty functions are almost identical. The above results indicate that the Lasso penalty function tends to shrink more parameters to zero, which helps us better identify important variables. However, it is also possible for this to be over-fitted, and this may cause more important variables to be ignored. Combining the above results leads to the following conclusions. First, it follows from a negative estimate of $\beta_{1}$ that there is a negative correlation between nodular echogenicity and malignant thyroid nodules, i.e., hypoechogenicity often indicates that the thyroid nodule is malignant. In addition, it follows from ${\hat{\beta }}_{8},{\hat{\beta }}_{13},{\hat{\beta }}_{14},{\hat{\beta }}_{26}$ that aspect ratio, margins, and microcalcifications are closely related to malignant thyroid nodules, which is consistent with the conclusion of [38]. Second, by ${\hat{\beta }}_{9}$ obtained with the SCAD penalty function, there is a positive correlation between age and malignant thyroid nodules. The coefficients associated with nodule size $x_{5}$, sex $x_{10}$, and internal structure (solidity $x_{16}$, cystic $x_{17}$, cystic solid mixture $x_{18}$) are shrunk to zero, implying that these covariates are not associated with malignant thyroid nodules, which is consistent with the conclusion regarding the role of predicting telomerase reverse transcriptase (TERT) promoter mutations in follicular thyroid cancer [39,40]. Third, there are correlations between certain data types, but their interaction effects have not been explored well, in that some of these data types represent certain interaction effects; for example, the aspect ratio represents the interaction effect between longitudinal diameter and horizontal diameter. By ${\hat{\beta }}_{6}$, ${\hat{\beta }}_{7}$, and ${\hat{\beta }}_{8}$, although longitudinal diameter $x_{6}$ and horizontal diameter $x_{7}$ are not correlated with malignant thyroid nodules, their interaction effect (aspect ratio $x_{8}$) has a negative effect on malignant thyroid nodules.

## 6. Discussion

This paper studies growing dimensional unconditional moment models in the presence of missing responses at random, constructs the modified unconditional moment models via the inverse probability weighted approach to deal with missing data, utilizes logistic regression models to describe propensity score functions, employs calibration conditions to establish estimating equations for estimating parameters in logistic regression models, and proposes a penalized log-ET likelihood ratio function to estimate unknown parameters in growing dimensional unconditional moment models and logistic regression models by combining the modified unconditional moment models and calibration conditions. To test contrasting hypotheses, we construct a constrainedly penalized ET likelihood ratio statistic, which is theoretically shown to asymptotically follow the centred chi-squared distribution that extends Wilks’ theory to growing unconditional moment models with missing response at random. Empirical results demonstrate that the proposed method has better estimation properties and more accurate variable selection than other methods, such as GMM and penalized empirical likelihood methods. Also, empirical results evidence that the proposed penalized ET likelihood method is robust to misspecified moment models. The thyroid data taken from the First People’s Hospital of Yunnan Province are used to illustrate the proposed method, and parameter estimates show the consistency with [38,39,40].

The proposed method has the following merits. First, it can simultaneously estimate unknown parameters associated with growing dimensional unconditional moment models and propensity score functions and select important variables. Second, novel unified equations are constructed to jointly rather than separately estimate unknown parameters by incorporating growing dimensional unconditional moment models and calibration conditions, which largely enhances the efficiency of the algorithm. Third, under some regularity conditions, we show the oracle property and asymptotic property of parameter estimators.

This paper focuses on missing at random of response variable and logistic regression models of propensity score functions. It is possible to extend the proposed method to nonignorable missing responses together with unknown forms of propensity score functions based on the neural network method to approximate the unknown propensity score functions.

## Figures and Tables

**Table 1:** Bias, SD, and RMSE values of non-zero parameters and performance of variable selection in Experiment 1.

		Bias			SD			RMSE							
(n,p+1)	Case	${\hat{\beta }}_{c}$	${\hat{\beta }}_{x,1}$	${\hat{\beta }}_{x,2}$	${\hat{\beta }}_{c}$	${\hat{\beta }}_{x,1}$	${\hat{\beta }}_{x,2}$	${\hat{\beta }}_{c}$	${\hat{\beta }}_{x,1}$	${\hat{\beta }}_{x,2}$	TP	FP	UF	CF	OF
(50,9)	M1	−0.0256	0.0811	−0.0285	0.841	0.863	0.313	0.842	0.867	0.315	5.779	0.141	0.196	0.697	0.107
	M2	−0.0465	−0.0255	0.1355	0.537	0.501	0.870	0.539	0.502	0.880	5.576	0.165	0.334	0.563	0.103
	M3	−0.0001	−0.0132	0.0427	0.851	0.793	0.822	0.851	0.793	0.823	5.689	0.139	0.262	0.639	0.099
(100,13)	M1	0.0464	−0.0228	−0.0195	0.750	0.146	0.135	0.751	0.148	0.137	9.785	0.101	0.174	0.752	0.074
	M2	−0.0714	−0.0357	−0.0548	0.378	0.741	0.708	0.384	0.742	0.710	9.584	0.142	0.297	0.611	0.092
	M3	0.0487	−0.0118	−0.0230	0.803	0.221	0.210	0.805	0.221	0.211	9.752	0.095	0.196	0.736	0.068
(200,17)	M1	−0.0175	−0.0044	−0.0042	0.153	0.088	0.088	0.154	0.088	0.089	13.905	0.039	0.082	0.881	0.037
	M2	−0.0293	−0.0193	−0.0158	0.186	0.141	0.125	0.188	0.143	0.126	13.811	0.081	0.137	0.794	0.069
	M3	−0.0234	−0.0288	−0.0081	0.168	0.218	0.118	0.169	0.220	0.118	13.850	0.068	0.116	0.825	0.059
(500,23)	M1	0.0000	0.0000	0.0001	0.005	0.005	0.005	0.005	0.005	0.005	19.975	0.000	0.022	0.978	0.000
	M2	−0.0050	0.0001	0.0014	0.094	0.084	0.077	0.094	0.084	0.077	19.929	0.015	0.061	0.927	0.012
	M3	−0.0003	−0.0004	0.0003	0.009	0.009	0.009	0.009	0.009	0.009	19.970	0.000	0.028	0.972	0.000
(500,101)	M1	−0.0004	−0.0005	−0.0001	0.050	0.010	0.010	0.050	0.010	0.010	97.934	0.000	0.040	0.960	0.000
	M2	−0.0068	−0.0013	−0.0036	0.096	0.073	0.069	0.096	0.074	0.069	97.703	0.024	0.145	0.834	0.021
	M3	−0.0044	0.0043	−0.0031	0.077	0.076	0.046	0.077	0.076	0.046	97.902	0.012	0.063	0.925	0.012
		**Bias**			**SD**			**RMSE**							
(n,q+1)	**Case**	${\hat{\gamma }}_{c}$	${\hat{\gamma }}_{u,1}$	${\hat{\gamma }}_{u,2}$	${\hat{\gamma }}_{c}$	${\hat{\gamma }}_{u,1}$	${\hat{\gamma }}_{u,2}$	${\hat{\gamma }}_{c}$	${\hat{\gamma }}_{u,1}$	${\hat{\gamma }}_{u,2}$	**TP**	**FP**	**UF**	**CF**	**OF**
(50,5)	M1	−0.0950	0.0261	−0.0159	0.369	0.786	0.296	0.381	0.786	0.297	1.905	0.176	0.093	0.747	0.160
	M2	−0.0680	−0.0516	−0.0246	0.570	0.526	0.513	0.574	0.528	0.513	1.864	0.174	0.128	0.732	0.140
	M3	0.0055	−0.0391	−0.0298	0.786	0.481	0.459	0.786	0.483	0.460	1.871	0.103	0.123	0.790	0.087
(100,7)	M1	−0.0410	0.0457	0.0849	0.229	0.795	0.791	0.232	0.796	0.796	3.923	0.112	0.075	0.825	0.100
	M2	−0.0368	0.0230	−0.0212	0.445	0.852	0.410	0.446	0.852	0.411	3.818	0.141	0.161	0.727	0.112
	M3	−0.0687	−0.1052	−0.0268	0.727	0.712	0.322	0.730	0.720	0.323	3.900	0.138	0.094	0.785	0.121
(200,9)	M1	−0.0263	−0.0240	−0.0074	0.199	0.138	0.163	0.200	0.140	0.163	5.955	0.066	0.042	0.893	0.065
	M2	−0.0324	0.0002	0.0036	0.556	0.551	0.544	0.556	0.551	0.544	5.920	0.062	0.072	0.873	0.055
	M3	−0.0229	−0.0188	−0.0089	0.175	0.148	0.110	0.177	0.150	0.110	5.954	0.066	0.040	0.899	0.061
(500,12)	M1	0.0001	−0.0005	−0.0000	0.006	0.006	0.006	0.006	0.006	0.006	8.981	0.000	0.019	0.981	0.000
	M2	−0.0143	−0.0147	−0.0127	0.119	0.111	0.080	0.120	0.112	0.081	8.971	0.058	0.028	0.917	0.055
	M3	−0.0002	0.0001	−0.0003	0.008	0.008	0.008	0.008	0.008	0.008	8.989	0.000	0.011	0.989	0.000
(500,51)	M1	−0.0195	−0.0138	−0.0090	0.134	0.107	0.065	0.135	0.108	0.065	47.957	0.052	0.035	0.915	0.050
	M2	−0.0068	−0.0073	−0.0049	0.092	0.092	0.060	0.093	0.092	0.060	47.862	0.026	0.083	0.894	0.023
	M3	−0.0173	−0.0160	−0.0023	0.168	0.109	0.053	0.169	0.110	0.053	47.900	0.032	0.070	0.901	0.029

**Table 2:** Bias, SD, and RMSE values of non-zero parameters and performance of variable selection in Experiment 2.

		Bias			SD			RMSE							
(n,p+1)	Case	${\hat{\beta }}_{c}$	${\hat{\beta }}_{x,1}$	${\hat{\beta }}_{x,2}$	${\hat{\beta }}_{c}$	${\hat{\beta }}_{x,1}$	${\hat{\beta }}_{x,2}$	${\hat{\beta }}_{c}$	${\hat{\beta }}_{x,1}$	${\hat{\beta }}_{x,2}$	TP	FP	UF	CF	OF
(50,9)	PETL	−0.0200	−0.0327	−0.0237	0.843	0.218	0.220	0.844	0.221	0.222	5.760	0.124	0.208	0.698	0.094
	HT	0.0013	−0.0486	0.0221	0.958	0.591	0.910	0.958	0.593	0.911	4.103	0.330	0.717	0.200	0.083
	ST	−0.0346	−0.0349	0.0358	0.923	0.848	0.884	0.924	0.849	0.885	2.961	0.222	0.854	0.114	0.032
	PLS	−0.1264	−0.0767	−0.0232	0.762	0.974	0.732	0.772	0.977	0.732	4.507	0.365	0.699	0.214	0.087
(100,13)	PETL	0.0477	−0.0238	−0.0195	0.749	0.227	0.218	0.750	0.228	0.219	9.792	0.089	0.167	0.767	0.066
	HT	−0.1052	−0.0493	0.0476	0.482	0.421	0.813	0.494	0.424	0.815	7.994	0.238	0.530	0.365	0.105
	ST	−0.0732	−0.0083	−0.0501	0.538	0.497	0.489	0.543	0.497	0.491	4.475	0.157	0.774	0.196	0.030
	PLS	−0.0250	−0.0364	0.0230	0.868	0.565	0.832	0.869	0.566	0.833	8.799	0.326	0.523	0.342	0.135
(200,17)	PETL	0.0063	0.0006	0.0017	0.165	0.081	0.080	0.165	0.081	0.080	13.909	0.000	0.074	0.926	−0.000
	HT	−0.0558	−0.0372	−0.0278	0.277	0.479	0.200	0.283	0.480	0.202	12.867	0.143	0.287	0.616	0.097
	ST	−0.0303	−0.0310	−0.0268	0.191	0.375	0.376	0.193	0.377	0.377	8.295	0.090	0.603	0.363	0.034
	PLS	−0.0275	−0.0138	−0.0102	0.182	0.136	0.123	0.184	0.137	0.123	13.485	0.083	0.206	0.734	0.060
(500,101)	PETL	0.0000	−0.0004	−0.0001	0.008	0.008	0.008	0.008	0.008	0.008	97.984	0.000	0.012	0.988	0.000
	HT	−0.0286	−0.0124	−0.0181	0.181	0.121	0.119	0.183	0.122	0.120	94.261	0.096	0.138	0.781	0.081
	ST	−0.0075	−0.0018	−0.0054	0.118	0.080	0.081	0.118	0.080	0.081	74.997	0.021	0.450	0.536	0.014
	PLS	−0.0537	−0.0404	−0.0346	0.236	0.158	0.200	0.242	0.163	0.203	95.283	0.195	0.305	0.565	0.130
		**Bias**			**SD**			**RMSE**							
(n,q+1)	**Case**	${\hat{\gamma }}_{c}$	${\hat{\gamma }}_{u,1}$	${\hat{\gamma }}_{u,2}$	${\hat{\gamma }}_{c}$	${\hat{\gamma }}_{u,1}$	${\hat{\gamma }}_{u,2}$	${\hat{\gamma }}_{c}$	${\hat{\gamma }}_{u,1}$	${\hat{\gamma }}_{u,2}$	**TP**	**FP**	**UF**	**CF**	**OF**
(50,5)	PETL	−0.0852	0.0307	−0.0106	0.331	0.785	0.247	0.342	0.785	0.247	1.904	0.152	0.094	0.768	0.138
	HT	−0.1180	−0.0948	−0.0058	0.622	0.565	0.694	0.633	0.572	0.694	1.332	0.331	0.505	0.342	0.153
	ST	−0.0448	−0.0687	−0.0474	0.885	0.683	0.649	0.886	0.687	0.650	1.076	0.201	0.691	0.250	0.059
	PLS	−0.1135	−0.0745	−0.0625	0.776	0.762	0.763	0.784	0.766	0.766	1.586	0.274	0.345	0.489	0.166
(100,7)	PETL	−0.0476	−0.0422	−0.0232	0.299	0.267	0.247	0.302	0.270	0.248	3.923	0.127	0.074	0.814	0.112
	HT	−0.0459	−0.0020	−0.0367	0.785	0.802	0.395	0.787	0.802	0.397	3.203	0.233	0.461	0.424	0.115
	ST	0.0010	−0.0369	−0.0412	0.810	0.800	0.485	0.810	0.801	0.487	1.767	0.191	0.765	0.201	0.034
	PLS	−0.1247	−0.1587	−0.1148	0.870	0.794	0.778	0.879	0.810	0.786	3.548	0.343	0.316	0.459	0.225
(200,9)	PETL	−0.0161	−0.0239	−0.0121	0.144	0.140	0.097	0.145	0.142	0.097	5.955	0.058	0.040	0.902	0.058
	HT	−0.0386	−0.0296	−0.0288	0.261	0.233	0.225	0.264	0.235	0.227	5.433	0.125	0.276	0.635	0.089
	ST	−0.0331	−0.0295	−0.0151	0.205	0.179	0.131	0.208	0.182	0.132	4.041	0.103	0.540	0.420	0.040
	PLS	−0.0366	−0.0219	−0.0215	0.207	0.164	0.132	0.210	0.166	0.133	5.772	0.106	0.150	0.764	0.086
(500,51)	PETL	−0.0009	0.0003	0.0021	0.009	0.009	0.033	0.009	0.009	0.033	47.991	0.000	0.007	0.993	0.000
	HT	−0.0259	−0.0283	−0.0235	0.167	0.161	0.113	0.169	0.163	0.116	46.242	0.108	0.127	0.779	0.094
	ST	−0.0366	−0.0220	−0.0045	0.334	0.132	0.082	0.336	0.134	0.082	35.787	0.052	0.447	0.527	0.026
	PLS	−0.0434	−0.0359	−0.0240	0.226	0.179	0.127	0.230	0.183	0.130	46.535	0.146	0.298	0.602	0.100

**Table 3:** Bias, SD, and RMSE values of parameter estimates for penalized ET likelihood (PETL), GMM, and PEL in Experiment 3.

		PETL					GMM					PEL				
(n,p)	Par	Bias	SD	RMSE	AL	CP	Bias	SD	RMSE	AL	CP	Bias	SD	RMSE	AL	CP
(117,8)	${\hat{\beta }}_{12}$	−0.0132	0.142	0.143	1.462	97.4	−0.0323	0.316	0.317	2.646	88.4	0.0065	0.549	0.549	1.279	81.2
	${\hat{\beta }}_{21}$	−0.0208	0.154	0.155	1.952	97.8	−0.0349	0.320	0.322	2.659	88.7	−0.0253	0.155	0.157	1.501	93.7
	${\hat{\beta }}_{32}$	−0.0233	0.161	0.162	1.945	97.5	−0.0245	0.331	0.332	1.197	81.0	−0.0229	0.151	0.152	1.269	93.5
(241,32)	${\hat{\beta }}_{12}$	−0.0064	0.069	0.069	0.878	97.1	−0.0319	0.262	0.264	2.912	91.6	−0.0101	0.105	0.105	1.312	93.5
	${\hat{\beta }}_{21}$	−0.0029	0.068	0.068	0.618	97.6	−0.0290	0.146	0.148	2.994	94.4	−0.0147	0.109	0.110	1.936	94.9
	${\hat{\beta }}_{23}$	−0.0043	0.066	0.066	0.612	96.8	−0.0360	0.160	0.164	2.945	92.5	−0.0135	0.208	0.208	1.960	96.2
	${\hat{\beta }}_{32}$	0.0032	0.174	0.174	1.177	97.7	−0.0214	0.259	0.259	1.981	94.4	−0.0095	0.099	0.099	1.908	93.8
	${\hat{\beta }}_{34}$	0.0000	0.172	0.172	0.884	95.7	−0.0332	0.149	0.153	1.959	93.2	−0.0131	0.114	0.115	1.885	92.5
	${\hat{\beta }}_{43}$	0.0103	0.177	0.178	1.178	97.6	−0.0242	0.267	0.268	1.942	92.5	−0.0034	0.232	0.232	1.935	94.8
	${\hat{\beta }}_{54}$	−0.0046	0.076	0.076	0.875	96.2	−0.0240	0.136	0.138	2.915	91.7	−0.0098	0.231	0.231	1.927	94.3
(602,72)	${\hat{\beta }}_{12}$	−0.0007	0.029	0.029	0.598	99.8	−0.0106	0.117	0.117	1.394	92.9	−0.0082	0.073	0.074	1.427	95.1
	${\hat{\beta }}_{21}$	0.0009	0.030	0.030	0.595	99.3	−0.0053	0.058	0.058	1.384	92.3	−0.0120	0.084	0.085	1.429	95.2
	${\hat{\beta }}_{23}$	0.0006	0.030	0.030	0.599	99.7	−0.0106	0.078	0.079	0.579	90.2	−0.0071	0.066	0.066	1.008	95.0
	${\hat{\beta }}_{32}$	−0.0001	0.029	0.029	0.442	98.1	−0.0059	0.061	0.061	0.584	91.8	−0.0084	0.073	0.074	0.999	93.7
	${\hat{\beta }}_{34}$	−0.0001	0.031	0.031	0.315	99.8	−0.0068	0.063	0.064	0.986	93.0	−0.0061	0.060	0.060	0.601	94.5
	${\hat{\beta }}_{43}$	0.0005	0.030	0.030	0.450	99.8	−0.0073	0.118	0.118	0.600	94.3	−0.0056	0.060	0.061	1.003	94.7
	${\hat{\beta }}_{45}$	0.0061	0.132	0.132	0.443	91.2	−0.0057	0.061	0.061	0.591	92.8	−0.0087	0.071	0.071	0.606	94.9
	${\hat{\beta }}_{54}$	−0.0021	0.129	0.129	0.439	91.4	−0.0082	0.069	0.069	1.426	95.1	−0.0072	0.066	0.066	1.009	95.0
	${\hat{\beta }}_{56}$	0.0010	0.030	0.030	0.439	97.5	−0.0069	0.063	0.064	0.990	93.4	−0.0083	0.073	0.074	1.003	94.2
	${\hat{\beta }}_{65}$	0.0033	0.029	0.030	0.442	98.3	−0.0042	0.051	0.051	1.386	92.4	−0.0044	0.050	0.050	1.440	96.0
	${\hat{\beta }}_{76}$	0.0006	0.029	0.029	0.440	98.1	−0.0075	0.066	0.066	0.994	93.4	−0.0047	0.057	0.057	1.007	95.2

**Table 4:** TP, FP, UF, CF, and OF values of variable selection for penalized ET likelihood (PETL), GMM, and PEL in Experiment 3.

	PETL					GMM					PEL				
(n,p,q)	TP	FP	UF	CF	OF	TP	FP	UF	CF	OF	TP	FP	UF	CF	OF
(117,8,2)	4.878	0.114	0.112	0.794	0.094	4.703	0.156	0.256	0.630	0.114	4.783	0.138	0.195	0.696	0.109
(241,32,3)	24.840	0.034	0.122	0.851	0.027	24.404	0.358	0.362	0.441	0.197	24.354	0.141	0.343	0.566	0.091
(602,72,4)	60.866	0.000	0.081	0.919	0.000	60.277	0.118	0.225	0.689	0.086	60.253	0.128	0.228	0.686	0.086

**Table 5:** PETL estimates (Est) and 95% confidence intervals (CI) of parameters under three penalty functions in real example.

	Lasso		ALasso		SCAD	
Par.	Est	CI	Est	CI	Est	CI
${\hat{\beta }}_{1}$	−2.864	(−3.515,−2.073)	−2.850	(−3.248,−2.455)	−2.929	(−3.223,−2.630)
${\hat{\beta }}_{2}$	0.000	(−0.744,0.771)	−0.195	(−0.869,0.375)	−0.012	(−0.116,0.081)
${\hat{\beta }}_{3}$	−0.082	(−0.927,0.801)	−0.046	(−0.256,0.152)	−0.134	(−0.241,−0.031)
${\hat{\beta }}_{4}$	0.000	(−0.523,0.584)	0.040	(−0.640,0.614)	0.084	(−0.435,0.599)
${\hat{\beta }}_{5}$	0.000	(−0.471,0.597)	0.000	(−0.656,0.600)	0.000	(−0.296,0.302)
${\hat{\beta }}_{6}$	0.000	(−0.699,0.801)	0.128	(−0.505,0.727)	0.111	(−0.191,0.406)
${\hat{\beta }}_{7}$	0.000	(−0.381,0.404)	0.282	(−0.282,0.871)	0.283	(−0.011,0.582)
${\hat{\beta }}_{8}$	−3.118	(−3.643,−2.520)	−3.104	(−3.728,−2.491)	−3.139	(−3.636,−2.629)
${\hat{\beta }}_{9}$	0.000	(−1.056,0.742)	0.429	(−0.223,1.006)	0.469	(0.169,0.769)
${\hat{\beta }}_{10}$	0.000	(−0.592,0.625)	0.000	(−0.416,0.407)	0.000	(−0.505,0.496)
${\hat{\beta }}_{11}$	5.920	(5.374,6.541)	5.801	(5.607,5.984)	5.865	(5.371,6.371)
${\hat{\beta }}_{12}$	6.205	(5.570,6.824)	6.343	(5.817,6.897)	6.207	(5.904,6.505)
${\hat{\beta }}_{13}$	4.952	(4.428,5.586)	4.893	(4.530,5.304)	4.939	(4.641,5.243)
${\hat{\beta }}_{14}$	4.284	(3.599,5.111)	4.338	(3.789,4.867)	4.481	(3.987,4.990)
${\hat{\beta }}_{15}$	5.617	(5.211,6.096)	5.777	(5.179,6.373)	5.727	(5.628,5.820)
${\hat{\beta }}_{16}$	0.000	(−0.789,0.741)	0.000	(−0.389,0.405)	0.000	(−0.300,0.306)
${\hat{\beta }}_{17}$	0.000	(−0.357,0.442)	0.000	(−0.433,0.393)	0.000	(−0.097,0.095)
${\hat{\beta }}_{18}$	0.000	(−0.770,0.797)	0.000	(−0.425,0.412)	0.000	(−0.527,0.497)
${\hat{\beta }}_{19}$	0.000	(−0.512,0.566)	0.000	(−0.615,0.584)	0.000	(−0.099,0.093)
${\hat{\beta }}_{20}$	1.633	(1.078,2.248)	1.231	(0.813,1.618)	1.230	(0.700,1.736)
${\hat{\beta }}_{21}$	0.000	(−0.784,0.797)	0.000	(−0.614,0.563)	0.000	(−0.103,0.100)
${\hat{\beta }}_{22}$	8.851	(8.037,9.757)	8.603	(7.998,9.211)	8.478	(8.173,8.781)
${\hat{\beta }}_{23}$	0.000	(−0.390,0.413)	2.993	(2.369,3.586)	2.774	(2.248,3.263)
${\hat{\beta }}_{24}$	0.000	(−0.363,0.467)	2.901	(2.345,3.468)	2.635	(2.112,3.134)
${\hat{\beta }}_{25}$	0.000	(−0.364,0.354)	3.118	(2.753,3.498)	2.971	(2.867,3.090)
${\hat{\beta }}_{26}$	5.647	(5.060,6.238)	5.452	(5.055,5.836)	5.461	(4.923,5.955)

## Data Availability

The datasets generated and analysed are available from the corresponding author on reasonable request.

## Appendix A. Proof of Theorems

In what follows, we will present details for the proofs of lemmas and theorems. For simplicity, let $P_{n}\left\{f\left(\alpha \right)\right\}=n^{-1}\sum_{i=1}^{n}f\left(Z_{i};\alpha \right)$, $M\left(\theta \right)=E\{\partial G\left(D_{i};\theta \right)/\partial \theta \}$, $\Sigma \left(\theta \right)=E[\left\{G\left(D_{i};\theta \right)-E\left[G\left(D_{i};\theta \right)\right]\right\}{\left\{G\left(D_{i};\theta \right)-E\left[G\left(D_{i};\theta \right)\right]\right\}}^{⊤}]$, $\Sigma =\Sigma (\theta_{0})$, $M=M(\theta_{0})$, $\upsilon_{i}=\lambda^{⊤}G\left(D_{i};\theta \right)$ for i=1,…,n, and $\bar{\lambda }=\arg\inf_{\lambda \in \Lambda_{n}}l_{n}\left(\lambda ,\theta \right)$, where $\Lambda_{n}=\{\lambda \in R^{r}:||\lambda ||\leq o_{p}\left(t^{-1/2}n^{-1/\iota }\right)\}$ for some positive ι>2, t=r+m is the number of the modified estimating equations, including the growing-dimensional unconditional moment model and calibration conditions.

**Proof** **of** **Lemma** **1.** Taking the Taylor expansion of $l(\bar{\lambda },\theta )$ at λ=0, we have

(A1) $$\begin{array}{cc} l(\bar{\lambda },\theta ) & =\frac{\partial l(0)}{\partial \upsilon_{i}}{\bar{\lambda }}^{⊤}P_{n}\left[G\left(\theta \right)\right]+\frac{1}{2}{\bar{\lambda }}^{⊤}\left\{\frac{1}{n}\sum_{i=1}^{n}\frac{\partial^{2}l\left({\dot{v}}^{⊤}G\left(D_{i};\theta \right)\right)}{\partial \upsilon_{i}^{2}}G^{⊤}\left(D_{i};\theta \right)G\left(D_{i};\theta \right)\right\}\bar{\lambda }\\ \; & \geq -||\bar{\lambda }||\cdot ||P_{n}\left[G\left(\theta \right)\right]||+C||\bar{\lambda }{\left|\right|}^{2}, \end{array}$$

where $P_{n}\left\{G\left(\theta \right)\right\}=n^{-1}\sum_{i=1}^{n}G\left(D_{i};\theta \right)$. On the other hand,

(A2) $$\begin{array}{c} l\left(\bar{\lambda },\theta \right)\leq l\left(0,\theta \right)=0, \end{array}$$

Combining (A1) and (A2) yields $\left|\right|\bar{\lambda }\left|\right|\leq \left|\right|P_{n}\left[G\left(\theta \right)\right]\left|\right|$. It follows from Assumptions 1, 2, and 4 that the estimating equations $G(D_{i};\theta )$ belong to the P-Glivenko–Cantelli class and the collection of components of $G(D_{i};\theta )$ belong to P-Donsker class; then,

(A3) $$\begin{array}{c} ||P_{n}\left[G\left(\theta \right)\right]-E\left[G\left(D_{i};\theta \right)\right]||=O_{p}\left(\sqrt{t/n}\right),\\ ||P_{n}\left[G\left(\theta \right)G^{⊤}\left(\theta \right)\right]-\Sigma \left(\theta \right)||=O_{p}\left(t/\sqrt{n}\right),\\ ||P_{n}\left[\frac{\partial G(\theta )}{\partial \theta }\right]-M\left(\theta \right)||=O_{p}\left(\sqrt{ts/n}\right). \end{array}$$

In addition, by the uniform modulus of continuity results, we have

(A4) $$\begin{array}{c} ||P_{n}\left[G\left(\theta \right)\right]-E\left[G\left(D_{i};\theta \right)\right]-P_{n}\left[G\left(\theta_{0}\right)\right]+E\left[G\left(D_{i};\theta_{0}\right)\right]||=o_{p}\left(\sqrt{t/n}\right), \end{array}$$

for estimating equations G(D;θ) uniformly in $\theta \in \Theta_{n}$. Combining Assumption 5 and Equation (A4) yields $\left|\right|P_{n}\left[G\left(\theta \right)\right]\left|\right|=O_{p}\left(\sqrt{t/n}\right)$ and $\left|\right|\bar{\lambda }\left|\right|=O_{p}\left(\sqrt{t/n}\right)=o_{p}\left(t^{-1/2}n^{-1/\iota }\right)$ for some positive ι>2. Therefore, $\bar{\lambda }\in \mathrm{int}\left(\Lambda_{n}\right)\subseteq {\hat{\Lambda }}_{n}\left(\theta \right)$, which indicates $\partial l(\bar{\lambda },\theta )/\partial \lambda =0$. By the convexity of $-l_{n}\left(\lambda ,\theta \right)$ and ${\hat{\Lambda }}_{n}\left(\theta \right)$, we have $\bar{\lambda }=\bar{\lambda }\left(\theta \right)$, and $\arg\inf_{\lambda \in {\hat{\Lambda }}_{n}\left(\theta \right)}l_{n}\left(\lambda ,\theta \right)$ exists. The first-order partial derivative of $l_{n}\left(\lambda ,\theta \right)$ with respect to λ is given by

$$\begin{array}{cc} \frac{\partial l_{n}\left(\lambda ,\theta \right)}{\partial \lambda }= & \frac{1}{n}\sum_{i=1}^{n}\frac{\exp[\lambda^{⊤}G\left(D_{i};\theta \right)]}{\frac{1}{n}\sum_{i=1}^{n}\exp\left[\lambda^{⊤}G\left(D_{i};\theta \right)\right]}G\left(D_{i};\theta \right)\\ = & \sum_{i=1}^{n}\frac{\exp\left[\lambda^{⊤}G\left(D_{i};\theta \right)\right]G\left(D_{i};\theta \right)}{\sum_{i=1}^{n}\exp\left[\lambda^{⊤}G\left(D_{i};\theta \right)\right]}\\ = & \sum_{i=1}^{n}\frac{\left[1+\lambda^{⊤}G\left(D_{i};\theta \right)\left(1+o_{p}\left(1\right)\right)\right]G\left(D_{i};\theta \right)}{\sum_{i=1}^{n}\exp\left[\lambda^{⊤}G\left(D_{i};\theta \right)\right]}=0, \end{array}$$

where the third equation holds due to $\max_{1\leq i\leq n}\sup_{\theta }\left|\lambda^{⊤}G\left(D_{i};\theta \right)\right|=o_{p}\left(1\right)$ for $\lambda \in \Lambda_{n}$. By Assumption 5 and Equation (A3), we have

$$\begin{array}{c} \bar{\lambda }\left(\theta \right)=-\Sigma^{-1}\left(\theta \right)P_{n}\left[G\left(\theta \right)\right]+o_{p}\left(\sqrt{t/n}\right), \end{array}$$

and then the ET likelihood $l_{n}\left(\lambda ,\theta \right)$ can be written as

(A5) $$\begin{array}{c} l_{n}\left(\lambda ,\theta \right)=-\frac{1}{2}P_{n}\left[G^{⊤}\left(\theta \right)\right]\Sigma^{-1}\left(\theta \right)P_{n}\left[G\left(\theta \right)\right]+o_{p}\left(t/n\right). \end{array}$$

Moreover, it follows from Equation (A3) that the expansion of $P_{n}\left[G\left(\theta \right)\right]$ at $\theta_{0}$ is given by

(A6) $$\begin{array}{c} P_{n}\left[G\left(\theta \right)\right]=P_{n}\left[G\left(\theta_{0}\right)\right]+M^{⊤}\left(\theta -\theta_{0}\right)+o_{p}\left(\sqrt{t/n}\right) \end{array}$$

for any $\theta \in D_{n\theta }$. Thus, combining Equations (A5) and (A6) yields

(A7) $$\begin{array}{cc} l_{n}\left(\lambda ,\theta \right)= & -\frac{1}{2}{\left(\theta -\theta_{0}\right)}^{⊤}M\Sigma^{-1}M^{⊤}\left(\theta -\theta_{0}\right)+{\left(\theta -\theta_{0}\right)}^{⊤}M\Sigma^{-1}P_{n}\left[G\left(\theta_{0}\right)\right]\\ & -\frac{1}{2}P_{n}\left[G^{⊤}\left(\theta_{0}\right)\right]\Sigma^{-1}P_{n}\left[G\left(\theta_{0}\right)\right]+o_{p}\left(t/n\right), \end{array}$$

and

(A8) $$\begin{array}{c} {\hat{\theta }}^{\mathrm{ET}}-\theta_{0}={\left(M\Sigma^{-1}M^{⊤}\right)}^{-1}M\Sigma^{-1}P_{n}\left[G\left(\theta_{0}\right)\right], \end{array}$$

where ${\hat{\theta }}^{\mathrm{ET}}$ is the ET likelihood estimator of θ. Combining Equations (A7) and (A8) yields

(A9) $$\begin{array}{cc} l_{n}\left(\lambda ,\theta \right)= & -\frac{1}{2}{\left(\theta -\theta_{0}\right)}^{⊤}M\Sigma^{-1}M^{⊤}\left(\theta -\theta_{0}\right)+{\left(\theta -\theta_{0}\right)}^{⊤}M\Sigma^{-1}M^{⊤}\left(\theta -\theta_{0}\right)\\ & -\frac{1}{2}P_{n}\left[G^{⊤}\left(\theta_{0}\right)\right]\Sigma^{-1}P_{n}\left[G\left(\theta_{0}\right)\right]+o_{p}\left(t/n\right)\\ = & -\frac{1}{2}{\left(\theta -\theta_{0}\right)}^{⊤}M\Sigma^{-1}M^{⊤}\left(\theta -2{\hat{\theta }}^{\mathrm{ET}}+\theta_{0}\right)\\ & -\frac{1}{2}P_{n}\left[G^{⊤}\left(\theta_{0}\right)\right]\Sigma^{-1}P_{n}\left[G\left(\theta_{0}\right)\right]+o_{p}\left(t/n\right)\\ = & -\frac{1}{2}{\left(\theta -{\hat{\theta }}^{\mathrm{ET}}\right)}^{⊤}M\Sigma^{-1}M^{⊤}\left(\theta -{\hat{\theta }}^{\mathrm{ET}}\right)+o_{p}\left(t/n\right). \end{array}$$

By the local quadratic approximation to the penalty function $p_{\nu }\left(\cdot \right)$, we obtain

(A10) $$\begin{array}{c} p_{\nu }\left(\theta \right)\propto \frac{1}{2}{\left(\theta -\theta_{0}\right)}^{⊤}V_{0}\left(\theta -\theta_{0}\right)+o_{p}\left(t/n\right). \end{array}$$

Combining Equations (A9) and (A10) yields

$$\begin{array}{cc} l_{pn}\left(\theta \right)= & l_{n}\left(\lambda ,\theta \right)-p_{\nu }\left(\theta \right)\\ = & -\frac{1}{2}{\left(\theta -{\hat{\theta }}^{\mathrm{ET}}\right)}^{⊤}M\Sigma^{-1}M^{⊤}\left(\theta -{\hat{\theta }}^{\mathrm{ET}}\right)-\frac{1}{2}{\left(\theta -\theta_{0}\right)}^{⊤}V_{0}\left(\theta -\theta_{0}\right)+R_{n}\\ = & -\frac{1}{2}{\left(\theta -\dot{\theta }\right)}^{⊤}V\left(\theta -\dot{\theta }\right)+C_{l_{pn}}+o_{p}\left(t/n\right), \end{array}$$

where $C_{l_{pn}}=-\theta_{0}^{⊤}V_{0}\theta_{0}-{\hat{\theta }}^{\mathrm{ET}}M\Sigma^{-1}M^{⊤}{\hat{\theta }}^{\mathrm{ET}}+{\dot{\theta }}^{⊤}J\dot{\theta }$ is some constant, $V=V_{0}+M\Sigma^{-1}M^{⊤}$, and $\dot{\theta }=V^{-1}\left(V_{0}\theta_{0}+M\Sigma^{-1}M^{⊤}{\hat{\theta }}^{\mathrm{ET}}\right)$. Hence, the penalized log-ET likelihood ratio function can be expressed as

$$l_{pn}\left(\theta \right)=-\frac{1}{2}{\left(\theta -\dot{\theta }\right)}^{⊤}V\left(\theta -\dot{\theta }\right)+o_{p}\left(t/n\right).$$

Thus, we complete the proof of Lemma 1. □

**Proof** **of** **Theorem** **1.** Let ${\overset{ˇ}{\theta }}^{\mathrm{PET}}={\left({\overset{ˇ}{\theta }}_{1}^{\mathrm{PET}⊤},{\overset{ˇ}{\theta }}_{2}^{\mathrm{PET}⊤}\right)}^{⊤}$ be the penalized ET likelihood estimator of θ when the model is correctly specified. (i) Firstly, we show that $\left|\right|{\overset{ˇ}{\theta }}_{1}^{\mathrm{PET}}-\theta_{10}\left|\right|\to 0$ as n→∞. Let $\theta^{*}={\left(\theta_{1}^{⊤},0\right)}^{⊤}$, for the constrained subspace $\left\{\theta \in R^{s}:\theta_{A_{\theta }^{c}}=0\right\}$, it follows from $p_{\nu }\left(0\right)=0$ and $L\left(\theta \right)=L\left(\theta^{*}\right)$ that $l_{pn}\left(\theta \right)=l_{pn}\left(\theta^{*}\right)$. Let ${\overset{ˇ}{\theta }}^{*}={\left({\overset{ˇ}{\theta }}_{1}^{\mathrm{PET}⊤},0\right)}^{⊤}$ be the maximizer of $l_{pn}\left(\theta \right)$ on the constrained subspace $\left\{\theta \in R^{s}:\theta_{A_{\theta }^{c}}=0\right\}$. Thus, it follows from the proof of Lemma 1 that $\left|\right|P_{n}\left[G\left({\overset{ˇ}{\theta }}^{*}\right)\right]\left|\right|=O_{p}\left(\sqrt{d_{\theta }/n}\right)$. Next, we draw the conclusion by contradiction trick, i.e., if $\left|\right|{\overset{ˇ}{\theta }}_{1}^{\mathrm{PET}}-\theta_{10}\left|\right|$ does not converge to zero in probability, there exists a subsequence $\left\{n_{f},d_{\theta f},t_{f}\right\}$, such that $\left|\right|{\overset{ˇ}{\theta }}_{1n_{f}}-\theta_{10}\left|\right|\geq \epsilon_{1}$ with probability tends to one for some constant $\epsilon_{1}>0$. On the other hand, from Assumption 6 we have $\lim\inf_{d_{\theta },t\to \infty }\psi_{1}\left(d_{\theta },t\right)>0$, and $||E\left[G\left(D_{i};{\overset{ˇ}{\theta }}_{n_{f}}^{*}\right)\right]\left|\right|=o_{p}\left\{\psi_{1}\left(d_{\theta f},t_{f}\right)\right\}+O_{p}\left(\sqrt{d_{\theta f}/n_{f}}\right)$, which is in conflict with $||E\left[G\left(D_{i};{\overset{ˇ}{\theta }}_{n_{f}}^{*}\right)\right]\left|\right|\geq \psi_{1}\left(d_{\theta f},t_{f}\right)\psi_{2}\left(\epsilon_{1}\right)$. Hence, it follows that $\left|\right|{\overset{ˇ}{\theta }}_{1}^{\mathrm{PET}}-\theta_{10}\left|\right|\to 0$ as n→∞. In addition, Assumption 4 indicates that $\left|\right|P_{n}\left[G\left({\overset{ˇ}{\theta }}^{*}\right)\right]-P_{n}\left[G\left(\theta_{0}\right)\right]\left|\right|\geq C||{\overset{ˇ}{\theta }}_{1}^{\mathrm{PET}}-\theta_{10}\left|\right|$ with probability tending to one for some constant *C*; thus, we have $\left|\right|{\overset{ˇ}{\theta }}_{1}^{\mathrm{PET}}-\theta_{10}\left|\right|=O_{p}\left(\sqrt{d_{\theta }/n}\right)$. Next, we show that ${\overset{ˇ}{\theta }}_{2}^{\mathrm{PET}}=0$ with probability tending to one. Let ${\overset{ˇ}{A}}_{\theta }=\left\{j:{\overset{ˇ}{\theta }}_{j}^{\mathrm{PET}}\neq 0\right\}$ be the index set of non-zero components of penalized ET likelihood estimator ${\overset{ˇ}{\theta }}^{\mathrm{PET}}$ under the correctly specified model, then its complement ${\overset{ˇ}{A}}_{\theta }^{c}=\left\{j:{\overset{ˇ}{\theta }}_{j}^{\mathrm{PET}}=0\right\}$. Hence, we need to show that $\mathrm{Pr}({\overset{ˇ}{A}}_{\theta }^{c}\neq A_{\theta }^{c})\to 0$ or $\mathrm{Pr}({\overset{ˇ}{A}}_{\theta }\neq A_{\theta })\to 0$ as n→∞. The tuning parameter ν in the penalty function controls the variable selection process to some extent; without losing generality, there exists a positive function η(ν) related to ν, such that ${\overset{ˇ}{A}}_{\theta }=\left\{j:\right|{\overset{ˇ}{\theta }}_{j}^{\mathrm{PET}}\left|>\eta \left(\nu \right)\right\}$. It is not hard to find that the event $\left\{{\overset{ˇ}{A}}_{\theta }\neq A_{\theta }\right\}$ is equivalent to the event $\left\{\right|{\overset{ˇ}{\theta }}_{j}^{\mathrm{PET}}|\leq \eta \left(\nu \right):\;\mathrm{some}\;j\in A_{\theta }\}⋃\{|{\overset{ˇ}{\theta }}_{j}^{\mathrm{PET}}\left|>\eta \left(\nu \right):\;\mathrm{some}\;j\in A_{\theta }^{c}\right\}$. Note that

$$\begin{array}{cc} \mathrm{Pr}(\{|{\overset{ˇ}{\theta }}_{j}^{\mathrm{PET}}|\leq \eta \left(\nu \right):\;\mathrm{some}\;j\in A_{\theta }\}) & \leq \sum_{j\in A_{\theta }}\mathrm{Pr}(|{\overset{ˇ}{\theta }}_{j}^{\mathrm{PET}}\left|\leq \eta \left(\nu \right)\right)\\ & =\sum_{j\in A_{\theta }}\mathrm{Pr}(|\theta_{0j}\left|-\right|{\overset{ˇ}{\theta }}_{j}^{\mathrm{PET}}\left|\geq \right|\theta_{0j}\left|-\eta \left(\nu \right)\right)\\ & \leq \sum_{j\in A_{\theta }}\mathrm{Pr}(|\theta_{0j}-{\overset{ˇ}{\theta }}_{j}^{\mathrm{PET}}\left|\geq \right|\theta_{0j}\left|-\eta \left(\nu \right)\right)\\ & \leq \sum_{j\in A_{\theta }}\mathrm{Pr}(|\theta_{0j}-{\overset{ˇ}{\theta }}_{j}^{\mathrm{PET}}|\geq \min_{j\in A_{\theta }}|\theta_{0j}\left|-\eta \left(\nu \right)\right)\\ & \leq \sum_{j\in A_{\theta }}\mathrm{Pr}(|{\overset{ˇ}{\theta }}_{j}^{\mathrm{PET}}-\theta_{0j}\left|\geq \eta \left(\nu \right)\right), \end{array}$$

where the last inequality holds due to Assumption 8. Similarly, we have $\mathrm{Pr}(\{|{\overset{ˇ}{\theta }}_{j}^{\mathrm{PET}}|>\eta \left(\nu \right):\;\mathrm{some}\;j\in A_{\theta }^{c}\})\leq \sum_{j\in A_{\theta }^{c}}\mathrm{Pr}(|{\overset{ˇ}{\theta }}_{j}^{\mathrm{PET}}-\theta_{0j}\left|\geq \eta \left(\nu \right)\right)$. Combining the above equations leads to $\mathrm{Pr}\left(\left\{{\overset{ˇ}{A}}_{\theta }\neq A_{\theta }\right\}\right)\leq \sum_{j=1}^{s}\mathrm{Pr}(|{\overset{ˇ}{\theta }}_{j}^{\mathrm{PET}}-\theta_{0j}\left|\geq \eta \left(\nu \right)\right)$. In addition, from the proof of Lemma 1, we have $\dot{\theta }={\left(M\Sigma^{-1}M^{⊤}+V_{0}\right)}^{-1}\left(M\Sigma^{-1}M^{⊤}{\overset{ˇ}{\theta }}^{\mathrm{PET}}\right)$ and ${\overset{ˇ}{\theta }}^{\mathrm{PET}}={\left(M\Sigma^{-1}M^{⊤}\right)}^{-1}M\Sigma^{-1}P_{n}\left[G\left(\theta_{0}\right)\right]+\theta_{0}$; it follows that

(A11) $$\begin{array}{c} \dot{\theta }-\theta_{0}=-{\left(M\Sigma^{-1}M^{⊤}+V_{0}\right)}^{-1}V_{0}\theta_{0}+{\left(M\Sigma^{-1}M^{⊤}+V_{0}\right)}^{-1}M\Sigma^{-1}P_{n}\left[G\left(\theta_{0}\right)\right], \end{array}$$

which yields $E\left[\dot{\theta }-\theta_{0}\right]=-{\left(M\Sigma^{-1}M^{⊤}+V_{0}\right)}^{-1}V_{0}\theta_{0}=\mu$ and $var\left[\dot{\theta }-\theta_{0}\right]={\left(M\Sigma^{-1}M^{⊤}+V_{0}\right)}^{-1}M\Sigma^{-1}M^{⊤}{\left(M\Sigma^{-1}M^{⊤}+V_{0}\right)}^{-1}=\sigma$. Let $\mu_{m}=\max_{j}\left\{\right|\mu_{j}\left|\right\}$ and $\sigma_{m}={\mathrm{EV}}_{\max}\left\{\sigma \right\}$, where ${\mathrm{EV}}_{\max}\left\{\sigma \right\}$ represents the maximum eigenvalue of σ, for some η(ν)>0,

$$\begin{array}{cc} & \sum_{j=1}^{s}\mathrm{Pr}(|{\overset{ˇ}{\theta }}_{j}^{\mathrm{PET}}-\theta_{0j}\left|\geq \eta \left(\nu \right)\right)\\ = & \sum_{j=1}^{s}\mathrm{Pr}((|{\overset{ˇ}{\theta }}_{j}^{\mathrm{PET}}-\theta_{0j}\left|-\right|\mu_{j}\left|\right)/\sqrt{\sigma_{m}}\geq \left(\eta \left(\nu \right)-\right|\mu_{j}\left|\right)/\sqrt{\sigma_{m}})\\ \leq & \sum_{j=1}^{s}\mathrm{Pr}((|{\overset{ˇ}{\theta }}_{j}^{\mathrm{PET}}-\theta_{0j}-\mu_{j}\left|\right)/\sqrt{\sigma_{jj}}\geq \left(\eta \left(\nu \right)-\mu_{j}\right)/\sqrt{\sigma_{m}})\\ \leq & s\mathrm{Pr}(|z|\geq \left(\eta \left(\nu \right)-\mu_{m}\right)/\sqrt{\sigma_{m}}), \end{array}$$

where $\mu_{j}$ is the *j*th diagonal element of μ and $\sigma_{jj}$ is the *j*th diagonal element of σ, and *z* is a standard normal random variable. By the Chebyshev inequality, we have

(A12) $$\begin{array}{cc} \sum_{j=1}^{s}\mathrm{Pr}(|{\overset{ˇ}{\theta }}_{j}^{\mathrm{PET}}-\theta_{0j}\left|\geq \eta \left(\nu \right)\right)\leq & \frac{s\sigma_{m}}{{\left(\eta \left(\nu \right)-\mu_{m}\right)}^{2}}. \end{array}$$

By some algebraic operations,

$$\begin{array}{cc} \sigma = & {\left(M\Sigma^{-1}M^{⊤}+V_{0}\right)}^{-1}M\Sigma^{-1}M^{⊤}{\left(M\Sigma^{-1}M^{⊤}+V_{0}\right)}^{-1}\\ = & {\left(M\Sigma^{-1}M^{⊤}+V_{0}\right)}^{-1}\left(M\Sigma^{-1}M^{⊤}+V_{0}-V_{0}\right){\left(M\Sigma^{-1}M^{⊤}+V_{0}\right)}^{-1}\\ = & {\left(M\Sigma^{-1}M^{⊤}+V_{0}\right)}^{-1}-V_{0}{\left(M\Sigma^{-1}M^{⊤}+V_{0}\right)}^{-2}. \end{array}$$

Thus, we have

$$\begin{array}{cc} \sigma_{m}= & {\mathrm{EV}}_{\max}\left\{{\left(M\Sigma^{-1}M^{⊤}+V_{0}\right)}^{-1}-V_{0}{\left(M\Sigma^{-1}M^{⊤}+V_{0}\right)}^{-2}\right\}\\ \leq & {\mathrm{EV}}_{\max}\left\{{\left(M\Sigma^{-1}M^{⊤}+V_{0}\right)}^{-1}\right\}\leq \varepsilon_{n}^{-1}, \end{array}$$

where $\varepsilon_{n}$ is the smallest eigenvalue of matrix $M\Sigma^{-1}M^{⊤}+\phi I$ and ϕ is a diagonal element of $V_{0}$. It follows from Assumptions 4 and 7 that $M\Sigma^{-1}M^{⊤}$ is a positive definite matrix, which leads to $\varepsilon_{n}\geq \phi$ and then $\sigma_{m}\leq \phi^{-1}$. Note that the *j*th diagonal element of matrix $V_{0}$ is $V_{0}^{kk}={\dot{p}}_{\nu }\left(\right|\theta_{k}^{\xi }\left|)/\right|\theta_{k}^{\xi }|$, where $\theta_{k}^{\xi }$ is a point lying in the line between $\theta_{k}$ and $\theta_{0k}$, for $\theta_{k}\in \{\theta_{k}:|\theta_{k}-\theta_{0k}\left|\leq C\sqrt{t/n}\right\}$. It follows from Assumption 8 that $V_{0}^{kk}={\dot{p}}_{\nu }\left(\right|\theta_{k}^{\xi }\left|)/\right|\theta_{k}^{\xi }|\propto {\dot{p}}_{\nu }\left(\right|\theta_{k}\left|\right)/\eta \left(\nu \right)$; hence, $\phi \geq \min_{k}V_{0}^{kk}=O\{\min_{k}{\dot{p}}_{\nu }\left(\right|\theta_{k}\left|)/\eta \left(\nu \right)\right\}$ and then $\sigma_{m}\leq O\{\eta \left(\nu \right)/\min_{k}{\dot{p}}_{\nu }\left(\right|\theta_{k}\left|)\right\}$. In addition, it is not difficult to obtain that

$$\begin{array}{cc} \mu_{m}= & ||{\left(M\Sigma^{-1}M^{⊤}+V_{0}\right)}^{-1}V_{0}\theta_{0}{\left|\right|}_{\infty }\\ = & ||{\left(M\Sigma^{-1}M^{⊤}+\phi I\right)}^{-1}\phi \theta_{0}{\left|\right|}_{\infty }, \end{array}$$

Implementing eigenvalue decomposition on matrix $M\Sigma^{-1}M^{⊤}$, we have $M\Sigma^{-1}M^{⊤}=QJQ^{⊤}$, where *Q* is an orthogonal matrix and $J=\mathrm{Diag}(j_{1},...,j_{s})$ is a diagonal matrix. Subsequently, $\mu_{m}=||Q\tilde{J}Q^{⊤}\theta_{0}{\left|\right|}_{\infty }$, where $\tilde{J}=\mathrm{Diag}\left(\frac{\phi }{j_{1}+\phi },...,\frac{\phi }{j_{s}+\phi }\right)$. Further, we obtain

$$\begin{array}{c} ||Q\tilde{J}Q^{⊤}\theta_{0}{\left|\right|}_{\infty }=||\tilde{J}\theta_{0}{\left|\right|}_{\infty }\leq \frac{\phi }{j^{*}+\phi }||\theta_{0}{\left|\right|}_{\infty }\leq \frac{\max_{k}V_{0}^{kk}}{j^{*}+\max_{k}V_{0}^{kk}}||\theta_{0}{\left|\right|}_{\infty }, \end{array}$$

where $j^{*}$ represents the minimum diagonal element of matrix $\tilde{J}$. Since all components of $\theta_{0}$ are finite and by Assumptions 8(ii) and 9(ii), we have $\mu_{m}\leq O\left\{\eta \left(\nu \right)\right\}$. Taking $\mu_{m}\leq O\left\{\eta \left(\nu \right)\right\}$ and $\sigma_{m}\leq O\{\eta \left(\nu \right)/\min_{k}{\dot{p}}_{\nu }\left(\right|\theta_{k}\left|)\right\}$ back to (A12) yields

(A13) $$\begin{array}{cc} & \sum_{j=1}^{s}\mathrm{Pr}(|{\overset{ˇ}{\theta }}_{j}^{\mathrm{PET}}-\theta_{0j}\left|\geq \eta \left(\nu \right)\right)\\ \leq & \frac{s\sigma_{m}}{{\left(\eta \left(\nu \right)-\mu_{m}\right)}^{2}}\\ \leq & s\frac{O\{\eta \left(\nu \right)/\min_{k}{\dot{p}}_{\nu }\left(\right|\theta_{k}\left|)\right\}}{{\left[\eta \left(\nu \right)-O\left\{\eta \left(\nu \right)\right\}\right]}^{2}}\\ \leq & O\left\{\frac{s}{\eta \left(\nu \right)\min_{k}{\dot{p}}_{\nu }\left(\right|\theta_{k}\left|\right)}\right\}. \end{array}$$

Combining Equation (A13) and Assumption 9(iii), we obtain that $\mathrm{Pr}(\left\{{\overset{ˇ}{A}}_{\theta }\neq A_{\theta }\right\})\to 0$ as n→∞, which indicates that ${\overset{ˇ}{\theta }}_{2}^{\mathrm{PET}}=0$ with probability tending to one. Below, we prove part (ii); let

$$\begin{array}{cc} H_{n1}\left(\lambda ,\theta \right)= & \partial l_{pn}\left(\lambda ,\theta \right)/\partial \lambda =P_{n}\left[\frac{\exp\{\lambda^{⊤}G\left(\theta \right)\}G\left(\theta \right)}{P_{n}\left[\exp\left\{\lambda^{⊤}G\left(\theta \right)\right\}\right]}\right],\\ H_{n2}\left(\lambda ,\theta \right)= & \partial l_{pn}\left(\lambda ,\theta \right)/\partial \theta =P_{n}\left[\frac{\exp\left\{\lambda^{⊤}G\left(\theta \right)\right\}{\left\{\partial_{\theta }G\left(\theta \right)\right\}}^{⊤}\lambda }{P_{n}\left[\exp\left\{\lambda^{⊤}G\left(\theta \right)\right\}\right]}\right], \end{array}$$

${\overset{ˇ}{\theta }}^{\mathrm{PET}}=\arg\max_{\theta \in \Theta }\inf_{\lambda \in {\hat{\Lambda }}_{n}\left(\theta \right)}l_{pn}\left(\theta \right)$ and ${\overset{ˇ}{\lambda }}^{\mathrm{PET}}=\arg\inf_{\lambda \in {\hat{\Lambda }}_{n}\left(\theta \right)}\max_{\theta \in \Theta }l_{pn}\left(\theta \right)$ under the correctly specified model; hence, $H_{n1}\left({\overset{ˇ}{\lambda }}^{\mathrm{PET}},{\overset{ˇ}{\theta }}^{\mathrm{PET}}\right)=0$ and $H_{n2}\left({\overset{ˇ}{\lambda }}^{\mathrm{PET}},{\overset{ˇ}{\theta }}^{\mathrm{PET}}\right)=0$. Subsequently, taking partial derivatives on function $H_{n1}\left(\lambda ,\theta \right)$ and $H_{n2}\left(\lambda ,\theta \right)$ for θ and λ, respectively, we have

$$\begin{array}{cc} H_{n11}\left(0,\theta_{0}\right)= & \partial H_{n1}\left(0,\theta_{0}\right)/\partial \lambda =P_{n}\left[G\left(\theta_{0}\right)G^{⊤}\left(\theta_{0}\right)\right]-P_{n}\left[G\left(\theta_{0}\right)\right]P_{n}{\left[G\left(\theta_{0}\right)\right]}^{⊤},\\ H_{n12}\left(0,\theta_{0}\right)= & \partial H_{n1}\left(0,\theta_{0}\right)/\partial \theta =P_{n}\left[\partial_{\theta }G\left(\theta_{0}\right)\right],\\ H_{n21}\left(0,\theta_{0}\right)= & \partial H_{n2}\left(0,\theta_{0}\right)/\partial \lambda =P_{n}{\left[\partial_{\theta }G\left(\theta_{0}\right)\right]}^{⊤},\\ H_{n22}\left(0,\theta_{0}\right)= & \partial H_{n2}\left(0,\theta_{0}\right)/\partial \theta =0. \end{array}$$

Taking Taylor’s expansion for $H_{n1}\left({\overset{ˇ}{\lambda }}^{\mathrm{PET}},{\overset{ˇ}{\theta }}^{\mathrm{PET}}\right)=0$ and $H_{n2}\left({\overset{ˇ}{\lambda }}^{\mathrm{PET}},{\overset{ˇ}{\theta }}^{\mathrm{PET}}\right)=0$ at $\left(0,\theta_{0}\right)$, yields

(A14) $$\left(\begin{array}{c} -H_{n1}\left(0,\theta_{0}\right)\\ 0 \end{array}\right)=\left(\begin{array}{cc} K_{1} & K_{2}\\ K_{2}^{⊤} & 0 \end{array}\right)\left(\begin{array}{c} {\overset{ˇ}{\lambda }}^{\mathrm{PET}}-0\\ {\overset{ˇ}{\theta }}^{\mathrm{PET}}-\theta_{0} \end{array}\right)+R_{n},$$

where $K_{1}=E\left\{P_{n}\left[G\left(\theta_{0}\right)G^{⊤}\left(\theta_{0}\right)\right]\right\}$, $K_{2}=E\left\{P_{n}\left[\partial_{\theta }G\left(\theta_{0}\right)\right]\right\}$, and $R_{n}=\sum_{k=1}^{5}R_{kn}$, $R_{n}={\left(R_{n}^{(1)⊤},R_{n}^{(2)⊤}\right)}^{⊤}$, $R_{1n}={\left(R_{1n}^{(1)⊤},R_{1n}^{(2)⊤}\right)}^{⊤}$, $R_{1n}^{\left(1\right)}={\left(R_{1n,1}^{\left(1\right)},\ldots ,R_{1n,t}^{\left(1\right)}\right)}^{⊤}$, $R_{1n}^{\left(2\right)}={\left(R_{1n,1}^{\left(2\right)},\ldots ,R_{1n,s}^{\left(2\right)}\right)}^{⊤}$,

$$\begin{array}{cc} R_{1n,j}^{\left(1\right)}= & \frac{1}{2}{\left({\overset{ˇ}{\kappa }}^{\mathrm{PET}}-\kappa_{0}\right)}^{⊤}\partial_{\kappa }^{2}H_{n1,j}\left(\kappa^{*}\right)\left({\overset{ˇ}{\kappa }}^{\mathrm{PET}}-\kappa_{0}\right),\;\mathrm{for}\;j=1,\ldots ,t\\ R_{1n,j}^{\left(2\right)}= & \frac{1}{2}{\left({\overset{ˇ}{\kappa }}^{\mathrm{PET}}-\kappa_{0}\right)}^{⊤}\partial_{\kappa }^{2}H_{n2,j}\left(\kappa^{*}\right)\left({\overset{ˇ}{\kappa }}^{\mathrm{PET}}-\kappa_{0}\right),\;\mathrm{for}\;j=1,\ldots ,s \end{array}$$

in which $\kappa ={\left(\lambda^{⊤},\theta^{⊤}\right)}^{⊤}$, $\partial_{\kappa }^{2}H_{nl,j}=\partial^{2}H_{nl,j}/\partial \kappa \partial \kappa^{⊤}$, and $H_{nl,j}$ represents the *j*th component of $H_{nl}$, l=1,2 and $\kappa^{*}={\left(\lambda^{*⊤},\theta^{*⊤}\right)}^{⊤}$, satisfying $\left|\right|\kappa^{*}-{\left(0,\theta_{0}^{⊤}\right)}^{⊤}\left|\right|\leq \left|\right|{\overset{ˇ}{\kappa }}^{\mathrm{PET}}-{\left(0,\theta_{0}^{⊤}\right)}^{⊤}\left|\right|$. Taking the Cauchy–Schwarz inequality to $R_{1n,j}^{\left(1\right)}$ yields

$$\begin{array}{cc} ||R_{1n,j}^{\left(1\right)}{\left|\right|}^{2}\leq & n^{-2}||{\overset{ˇ}{\kappa }}^{\mathrm{PET}}-\kappa_{0}{\left|\right|}^{4}\sum_{i,k}^{s+t}\partial^{2}H_{n1,j}/\partial \kappa \partial \kappa^{⊤},\\ = & O_{p}\left(t^{2}{\left(s+t\right)}^{2}n^{-2}\right) \end{array}$$

for j=1,...,s+t; it follows from Assumption 5 that $\left|\right|R_{1n,j}^{\left(1\right)}{\left|\right|}^{2}=o_{p}\left(1/n\right)$. The same operation applies to $R_{1n,j}^{\left(2\right)}$; we obtain $\left|\right|R_{1n,j}^{\left(2\right)}{\left|\right|}^{2}=o_{p}\left(1/n\right)$, which leads to $\left|\right|R_{1n,j}{\left|\right|}^{2}=o_{p}\left(1/n\right)$. Furthermore, there are $\left|\right|R_{1n}\left|\right|=o_{p}\left(\sqrt{1/n}\right)$. Let $W\left(\theta \right)=\{{\dot{p}}_{\nu }\left(\right|\theta_{1}|)\mathrm{sign}\left(\theta_{1}\right),{\dot{p}}_{\nu }\left(\right|\theta_{2}\left|\right)\mathrm{sign}\left(\theta_{2}\right),...,$${\dot{p}}_{\nu }\left(\right|\theta_{s}|)\mathrm{sign}\left(\theta_{s}\right)\}$; it follows from Assumption 9(i) that $\left|\right|R_{2n}\left|\right|=\left|\right|{\left(0,W^{⊤}\left(\theta_{0}\right)\right)}^{⊤}\left|\right|=o_{p}\left(\sqrt{1/n}\right)$, and $\left|\right|R_{3n}\left|\right|=\left|\right|{\left(0,\dot{W}\left(\theta^{\varrho }\right)\left({\overset{ˇ}{\theta }}^{\mathrm{PET}}-\theta_{0}\right)\right)}^{⊤}\left|\right|=o_{p}\left(\sqrt{1/n}\right)$, where $\dot{W}\left(\cdot \right)$ is the first-order derivatives of W(·) and $\theta^{\varrho }$ is a vector lying in the line between ${\overset{ˇ}{\theta }}^{\mathrm{PET}}$ and $\theta_{0}$. By Assumptions 4 and 5, we have $\left|\right|R_{4n}\left|\right|=\left|\right|{\left({\left\{\left(P_{n}\left[G\left(\theta_{0}\right)G^{⊤}\left(\theta_{0}\right)\right]-K_{1}\right){\overset{ˇ}{\lambda }}^{\mathrm{PET}}\right\}}^{⊤}+{\left\{\left(P_{n}\left[\partial_{\theta }G\left(\theta_{0}\right)\right]-K_{2}\right)\left({\overset{ˇ}{\theta }}^{\mathrm{PET}}-\theta \right)\right\}}^{⊤},0\right)}^{⊤}\left|\right|=o_{p}\left(\sqrt{1/n}\right)$ and $\left|\right|R_{5n}\left|\right|=\left|\right|{\left(0,{\overset{ˇ}{\lambda }}^{\mathrm{PET}⊤}P_{n}\left[\partial_{\theta }G\left(\theta_{0}\right)\right]-K_{2}\right)}^{⊤}\left|\right|=o_{p}\left(\sqrt{1/n}\right)$. Combining the above equations yields $\left|\right|R_{n}\left|\right|=o_{p}\left(\sqrt{1/n}\right)$. By Equation (A14), we have

(A15) $$\left(\begin{array}{c} {\overset{ˇ}{\lambda }}^{\mathrm{PET}}-0\\ {\overset{ˇ}{\theta }}^{\mathrm{PET}}-\theta_{0} \end{array}\right)=S^{-1}\left\{\left(\begin{array}{c} -H_{n1}\left(0,\theta_{0}\right)\\ 0 \end{array}\right)+R_{n}\right\}.$$

where

$$\begin{array}{c} S=\left(\begin{array}{cc} K_{1} & K_{2}\\ K_{2}^{⊤} & 0 \end{array}\right). \end{array}$$

Inverse the block matrix *S* and let

$$\begin{array}{c} S^{-1}=\left(\begin{array}{cc} S_{11} & S_{12}\\ S_{21} & S_{22} \end{array}\right) \end{array}$$

where

$$\begin{array}{cc} S_{11}= & K_{1}^{-1}-K_{1}^{-1}K_{2}{\left(K_{2}^{⊤}K_{1}^{-1}K_{2}\right)}^{-1}K_{2}^{⊤}K_{1}^{-1},\\ S_{12}= & K_{1}^{-1}K_{2}{\left(K_{2}^{⊤}K_{1}^{-1}K_{2}\right)}^{-1},\\ S_{21}= & {\left(K_{2}^{⊤}K_{1}^{-1}K_{2}\right)}^{-1}K_{2}^{⊤}K_{1}^{-1},\\ S_{22}= & -{\left(K_{2}^{⊤}K_{1}^{-1}K_{2}\right)}^{-1}, \end{array}$$

It follows that

(A16) $$\begin{array}{c} {\overset{ˇ}{\theta }}^{\mathrm{PET}}-\theta_{0}=-{\left(K_{2}^{⊤}K_{1}^{-1}K_{2}\right)}^{-1}K_{2}^{⊤}K_{1}^{-1}\left\{H_{n1}\left(0,\theta_{0}\right)+R_{n}^{\left(2\right)}\right\}, \end{array}$$

where $H_{n1}\left(0,\theta_{0}\right)=P_{n}\left[G\left(\theta \right)\right]$ and $\left|\right|R_{n}^{\left(2\right)}\left|\right|=o_{p}\left(\sqrt{1/n}\right)$. Let $\Gamma_{1}={\left(K_{2}^{⊤}K_{1}^{-1}K_{2}\right)}^{-1}$, $\Gamma_{2}=-\Gamma_{1}K_{2}^{⊤}K_{1}^{-1}$, and $M_{ni}=n^{-1/2}A_{n}\Gamma_{1}^{-1/2}\Gamma_{2}G\left(X_{i},Y_{i};\theta_{0}\right)$. For any $\epsilon_{2}>0$, by the Cauchy–Schwarz inequality,

$$\begin{array}{cc} \sum_{i=1}^{n}E[||M_{ni}{\left|\right|}^{2}]I(||M_{ni}||>\epsilon_{2})= & nE[||M_{ni}{\left|\right|}^{2}]I(||M_{ni}||>\epsilon_{2})\\ \leq & n\{E||M_{ni}{\left|\right|}^{4}{\}}^{1/2}\{\mathrm{Pr}(||M_{ni}||>\epsilon_{2}{)\}}^{1/2}. \end{array}$$

Since $A_{n}A_{n}^{⊤}\to V$, then $\mathrm{Pr}(||M_{ni}||>\epsilon_{2})\leq E[||M_{ni}{\left|\right|}^{2}]/\epsilon_{2}^{2}=O_{p}\left(n^{-1}\right)$ by the Markov inequality. Moreover,

$$\begin{array}{cc} E[||M_{ni}|{|}^{4}]= & \frac{1}{n^{2}}E{\left[G^{⊤}\left(X_{i},Y_{i};\theta \right)\Gamma_{2}\Gamma_{1}^{-1/2}A_{n}^{⊤}A_{n}\Gamma_{1}^{-1/2}\Gamma_{2}^{⊤}G\left(X_{i},Y_{i};\theta \right)\right]}^{2}\\ \leq & \frac{1}{n^{2}}E_{\max}^{2}\left(A_{n}^{⊤}A_{n}\right)E_{\max}^{2}\left(\Gamma_{1}^{-1}\right)E{\left[G^{⊤}\left(X_{i},Y_{i};\theta \right)G\left(X_{i},Y_{i};\theta \right)\right]}^{2}\\ = & O_{p}\left(s^{2}n^{-2}\right), \end{array}$$

which leads to $\sum_{i=1}^{n}E[||M_{ni}{\left|\right|}^{2}]I(||M_{ni}||>\epsilon_{2})=O_{p}\left(n^{-1/2}\right)=o_{p}\left(1\right)$. Furthermore, $\sum_{i=1}^{n}\mathrm{cov}\left(M_{ni}\right)=n\mathrm{cov}\left(M_{ni}\right)=A_{n}A_{n}^{⊤}\to V$ as n→∞ and $\Gamma_{2}H_{n1}\left(0,\theta_{0}\right)={\overset{ˇ}{\theta }}^{\mathrm{PET}}-\theta_{0}$. By the Lindeberg–Feller condition, we have $\sqrt{n}A_{n}\Gamma_{1}^{-1/2}\left({\overset{ˇ}{\theta }}^{\mathrm{PET}}-\theta_{0}\right)\overset{L}{⟶}N\left(0,V\right)$. □

**Proof** **of** **Theorem** **2.** Let ${\hat{\theta }}^{\mathrm{PET}}={\left({\hat{\theta }}_{1}^{\mathrm{PET}⊤},{\hat{\theta }}_{2}^{\mathrm{PET}⊤}\right)}^{⊤}$ be the penalized ET likelihood estimator of θ in the presence of EE misspecification. (i) By Assumptions 1, 2, and 4 and Equations (3) and (4), we have that $G(D_{i};\theta )$ is continuous in θ and $\sup_{j}E{\left[G_{j}\left(D_{i};\theta \right)\right]}^{2}\leq C_{1}^{G}$; then, $\sup_{\theta \in \Theta }||G\left(D_{i};{\hat{\theta }}^{\mathrm{PET}}\right)-E\left[G\left(D_{i};\theta \right)\right]\left|\right|\overset{P}{⟶}0$, where $\overset{P}{⟶}$ denotes convergence in probability. Subsequently, it follows from Assumptions 10–12 and 14 and Equations (3), (4), and (8) that ${\hat{\theta }}^{\mathrm{PET}}\overset{P}{⟶}\theta^{*}$. (ii) The first-order partial derivative of the penalized ET likelihood $l_{pn}\left(\theta \right)$ with respect to $\theta_{j}$ for $j\notin A_{\theta }$ is given by

(A17) $$\begin{array}{cc} \frac{\partial l_{pn}\left(\theta \right)}{\partial \theta_{j}}= & \frac{P_{n}\left[\exp\left\{\lambda^{⊤}G\left(\theta \right)\right\}\partial_{\theta_{j}}G^{⊤}\left(\theta \right)\right]}{P_{n}\left[\exp\left\{\lambda^{⊤}G\left(\theta \right)\right\}\right]}\lambda -{\dot{p}}_{\nu }\left(\right|\theta_{j}\left|\right)\mathrm{sign}\left(\theta_{j}\right)\\ = & \frac{E\left[\exp\left\{\lambda^{⊤}G\left(\theta \right)\right\}\partial_{\theta_{j}}G^{⊤}\left(\theta \right)\right]+O_{p}\left(\sqrt{1/n}\right)}{E\left[\exp\left\{\lambda^{⊤}G\left(\theta \right)\right\}\right]+O_{p}\left(\sqrt{1/n}\right)}\lambda -{\dot{p}}_{\nu }\left(\right|\theta_{j}\left|\right)\mathrm{sign}\left(\theta_{j}\right)\\ \leq & O_{p}\left(1\right)||\lambda ||-{\dot{p}}_{\nu }\left(\right|\theta_{j}\left|\right)\mathrm{sign}\left(\theta_{j}\right)\\ = & \nu \{\frac{||\lambda ||}{\nu }O_{p}\left(1\right)-\frac{{\dot{p}}_{\nu }\left(\right|\theta_{j}\left|\right)}{\nu }\mathrm{sign}\left(\theta_{j}\right)\}\\ = & \nu \{-\frac{{\dot{p}}_{\nu }\left(\right|\theta_{j}\left|\right)}{\nu }\mathrm{sign}\left(\theta_{j}\right)+o_{p}\left(1\right)\}, \end{array}$$

where the third inequality above is derived from Assumptions 13 and 14. It is not difficult to notice that the sign of $\partial l_{pn}\left(\theta \right)/\partial \theta_{j}$ is dominated by the sign of $\theta_{j}$ from Equation (A17). In other words, we have $\partial l_{pn}\left(\theta \right)/\partial \theta_{j}>0$ when $\theta_{j}<0$, and $\partial l_{pn}\left(\theta \right)/\partial \theta_{j}<0$ when $\theta_{j}>0$ w.p.1, as n→∞ for any $j\notin A_{\theta }$; therefore, ${\hat{\theta }}_{2}^{\mathrm{PET}}=0$ with probability tending to one. (iii) Denote $M_{1}$ and $M_{2}$ as a projection matrix, such that $M_{1}\theta =\theta_{1}$ and $M_{2}\theta =\theta_{2}$, then the penalized log-ET likelihood ratio function under constraint $M_{2}\theta =\theta_{2}=0$ is given by

$$S_{n}\left(\lambda ,\theta ,\tau \right)=\log P_{n}\left[\exp\left\{\lambda^{⊤}G\left(D_{i};\theta \right)\right\}\right]-\sum_{j=1}^{s}p_{\nu }\left(\right|\theta_{j}\left|\right)+\tau^{⊤}M_{2}\theta .$$

Taking partial derivatives of $S_{n}\left(\lambda ,\theta ,\tau \right)$ with respect to λ, θ, and τ as follows, respectively,

$$\begin{array}{cc} S_{n1}\left(\lambda ,\theta ,\tau \right)= & \partial S_{n}\left(\lambda ,\theta ,\tau \right)/\partial \lambda =P_{n}\left[\frac{\exp\left\{\lambda^{⊤}G\left(D_{i};\theta \right)\right\}G\left(D_{i};\theta \right)}{P_{n}\left[\exp\left\{\lambda^{⊤}G\left(D_{i};\theta \right)\right\}\right]}\right]\\ S_{n2}\left(\lambda ,\theta ,\tau \right)= & \partial S_{n}\left(\lambda ,\theta ,\tau \right)/\partial \theta =P_{n}\left[\frac{\exp\left\{\lambda^{⊤}G\left(D_{i};\theta \right)\right\}\partial_{\theta }G^{⊤}\left(D_{i};\theta \right)\lambda }{P_{n}\left[\exp\left\{\lambda^{⊤}G\left(D_{i};\theta \right)\right\}\right]}\right]+\omega \left(\theta \right)+M_{2}^{⊤}\tau \\ S_{n3}\left(\lambda ,\theta ,\tau \right)= & \partial S_{n}\left(\lambda ,\theta ,\tau \right)/\partial \tau =M_{2}\theta , \end{array}$$

where $W\left(\theta \right)=\{{\dot{p}}_{\nu }\left(\right|\theta_{1}|)\mathrm{sign}\left(\theta_{1}\right),{\dot{p}}_{\nu }\left(\right|\theta_{2}|)\mathrm{sign}\left(\theta_{2}\right),...,{\dot{p}}_{\nu }\left(\right|\theta_{s}|)\mathrm{sign}\left(\theta_{s}\right)\}$. Let ${\hat{\zeta }}^{\mathrm{PET}}={\left({\hat{\lambda }}^{\mathrm{PET}⊤},{\hat{\theta }}^{\mathrm{PET}⊤},{\hat{\tau }}^{\mathrm{PET}⊤}\right)}^{⊤}$ be the penalized ET estimator of $\zeta ={\left(\lambda^{⊤},\theta^{⊤},\tau^{⊤}\right)}^{⊤}$, which is the solution to the functions $S_{n1}\left(\lambda ,\theta ,\tau \right)$, $S_{n2}\left(\lambda ,\theta ,\tau \right)$, and $S_{n3}\left(\lambda ,\theta ,\tau \right)$. Subsequently, taking second partial derivatives of $S_{n}\left(\lambda ,\theta ,\tau \right)$ with respect to ζ, it is not hard to notice that the component of the second partial derivatives of $S_{n}\left(\lambda ,\theta ,\tau \right)$ is given by $\tilde{\zeta }\exp\left\{l_{1}\lambda^{⊤}G\left(D_{i};\theta \right)\right\}G^{l_{2}}{\left(\partial_{\theta }G\right)}^{l_{3}}{\left(\partial_{\theta_{j_{1}}\theta_{j_{2}}}G\right)}^{l_{4}}$, for $l_{1}=0,1$, $j_{1},j_{2}=1,...,s$, and $0\leq l_{2}+l_{3}+l_{4}\leq 2$, where *G*, $\partial_{\theta }G$, $\partial_{\theta_{j_{1}}\theta_{j_{2}}}G$ denote the combination of components of $G(D_{i};\theta )$, $\partial_{\theta }G\left(D_{i};\theta \right)$, $\partial_{\theta_{j_{1}}\theta_{j_{2}}}G\left(D_{i};\theta \right)$, respectively, and $\tilde{\zeta }$ denotes the product of elements of ζ. Let matrix $\tilde{S}\left(\lambda ,\theta ,\tau \right)=\left(S_{n1}\left(\lambda ,\theta ,\tau \right),S_{n2}\left(\lambda ,\theta ,\tau \right),S_{n3}\left(\lambda ,\theta ,\tau \right)\right)$; it follows that

(A18) $$\begin{array}{c} E[\underset{\zeta \in N_{\zeta }^{*}}{\sup}||\partial \tilde{S}\left(\lambda ,\theta ,\tau \right)/\partial \zeta ||]<\infty , \end{array}$$

from Assumption 13, where $N_{\zeta }^{*}$ is the neighbourhood of $\zeta^{*}={\left(\lambda^{*⊤},\theta^{*⊤},\tau^{*⊤}\right)}^{⊤}]$. In addition, the expectation

$$\begin{array}{cc} & E[\underset{\zeta \in N_{\zeta }^{*}}{\sup}\tilde{\zeta }\exp\left\{l_{1}\lambda^{⊤}G\left(D_{i};\theta \right)\right\}|G{|}^{l_{2}}|\partial_{\theta }G{|}^{l_{3}}|\partial_{\theta_{j_{1}}\theta_{j_{2}}}G{|}^{l_{4}}]\\ \leq & E[\underset{\zeta \in N_{\zeta }^{*}}{\sup}\tilde{\zeta }\exp\left\{l_{1}\lambda^{⊤}G\left(D_{i};\theta \right)\right\}|h\left(D_{i}\right){|}^{l_{2}}]\\ = & E[\underset{\zeta \in N_{\zeta }^{*}}{\sup}\tilde{\zeta }\exp\left\{l_{1}\lambda^{⊤}G\left(D_{i};\theta \right)\right\}h^{l_{2}}\left(D_{i}\right)]<\infty , \end{array}$$

where the second and fourth inequality above is due to Assumptions 10–13. The elements of the matrix $\tilde{S}\left(\lambda ,\theta ,\tau \right){\tilde{S}}^{⊤}\left(\lambda ,\theta ,\tau \right)$ are given by $\tilde{\zeta }\exp\left\{l_{1}\lambda^{⊤}G\left(D_{i};\theta \right)\right\}G^{l_{2}}{\left(\partial_{\theta }G\right)}^{l_{3}}$${\left(\partial_{\theta_{j_{1}}\theta_{j_{2}}}G\right)}^{l_{4}}$; hence

(A19) $$\begin{array}{c} E[\tilde{S}\left(\lambda ,\theta ,\tau \right){\tilde{S}}^{⊤}\left(\lambda ,\theta ,\tau \right)]<\infty . \end{array}$$

Combining inequalities (A18) and (A19) and Theorem 3.4 of Newey and McFadden (1994), we have $\sqrt{n}\left({\hat{\zeta }}^{\mathrm{PET}}-\zeta^{*}\right)\overset{L}{⟶}N\left(0,\Delta^{-1}\Omega \Delta^{-⊤}\right)$, where $\Delta =E[\partial_{\zeta }\tilde{S}\left(\lambda ,\theta ,\tau \right){|}_{\zeta =\zeta^{*}}]$, $\Omega =E[\tilde{S}\left(\lambda^{*},\theta^{*},\tau^{*}\right){\tilde{S}}^{⊤}\left(\lambda^{*},\theta^{*},\tau^{*}\right)]$, and $\overset{L}{⟶}$ denotes convergence in distribution. □

**Proof** **of** **Theorem** **3.** Being analogue to the proof of Theorem 1, we obtain that

$$\begin{array}{cc} & {\hat{\lambda }}^{\mathrm{PET}}=\left\{K_{1}^{-1}-K_{1}^{-1}K_{2}\Gamma_{1}K_{2}^{⊤}K_{1}^{-1}\right\}P_{n}\left[G\left(\theta \right)\right]+{\tilde{R}}_{n}^{\left(1\right)},\\ & {\hat{\theta }}^{\mathrm{PET}}-\theta_{0}=-\Gamma_{1}K_{2}^{⊤}K_{1}^{-1}P_{n}\left[G\left(\theta \right)\right]+{\tilde{R}}_{n}^{\left(2\right)}, \end{array}$$

where $\left|\right|{\tilde{R}}_{n}^{\left(1\right)}\left|\right|=\left|\right|{\tilde{R}}_{n}^{\left(2\right)}\left|\right|=o_{p}\left(\sqrt{1/n}\right)$. Taking the Taylor expansion of $l_{n}\left(\lambda ,\theta \right)$ at ${\hat{\omega }}_{i}={\hat{\lambda }}^{\mathrm{PET}⊤}G\left(X_{i},Y_{i};{\hat{\theta }}^{\mathrm{PET}}\right)$ yields $l_{n}\left(\lambda ,\theta \right)=P_{n}\left[\hat{\omega }\left(1+o_{p}\left(1\right)\right)\right]$. Substituting ${\hat{\lambda }}^{\mathrm{PET}}$ and ${\hat{\theta }}^{\mathrm{PET}}$ into $l_{n}\left(\lambda ,\theta \right)$ yields $2nl_{n}\left({\hat{\lambda }}^{\mathrm{PET}},{\hat{\theta }}^{\mathrm{PET}}\right)=-nP_{n}\left[G^{⊤}\left(\theta \right)\right]\left\{K_{1}^{-1}-K_{1}^{-1}K_{2}\Gamma_{1}K_{2}^{⊤}K_{1}^{-1}\right\}P_{n}\left[G\left(\theta \right)\right]+o_{p}\left(1\right)$. Let $\overset{˘}{\lambda }$ and $\overset{˘}{\theta }$ be the maximizing $l_{pn}\left(\theta \right)$ under null hypothesis $H_{0}:C_{n}\theta =0$. The constrained penalized ET likelihood under null hypothesis $H_{0}$ is given by

$$l_{cpn}\left(\lambda ,\theta ,\upsilon \right)=\log P_{n}\left[\exp\left\{\lambda^{⊤}G\left(\theta \right)\right\}\right]-\sum_{j=1}^{s}p_{\nu }\left(\right|\theta_{j}\left|\right)+\upsilon^{⊤}C_{n}\theta ,$$

where υ is a Lagrange multiplier. Since $C_{n}\theta =0$ and $C_{n}C_{n}^{⊤}=I_{e}$, through some algebraic operations, we obtain

$$\overset{˘}{\lambda }=\left\{K_{1}^{-1}-K_{1}^{-1}K_{2}\Gamma_{3}K_{2}^{⊤}K_{1}^{-1}\right\}P_{n}\left[G\left(\theta \right)\right]+{\overset{˘}{R}}_{n1},$$

where $\Gamma_{3}=\Gamma_{1}C_{n}^{⊤}{\left(C_{n}\Gamma_{1}C_{n}^{⊤}\right)}^{⊤}C_{n}\Gamma_{1}-\Gamma_{1}$. Subsequently, the constrained penalized ET likelihood is given by $2nl_{cpn}\left(\overset{˘}{\lambda },\overset{˘}{\theta }\right)=-nP_{n}\left[G^{⊤}\left(\theta \right)\right]\left\{K_{1}^{-1}-K_{1}^{-1}K_{2}\Gamma_{3}K_{2}^{⊤}K_{1}^{-1}\right\}P_{n}\left[G\left(\theta \right)\right]+o_{p}\left(1\right)$, where $o_{p}\left(1\right)$ includes a penalty function term. Combining the above equation, we obtain the constrained penalized ET likelihood ratio statistic under null hypothesis $H_{0}$ as follows:

(A20) $$\begin{array}{cc} \tilde{l}\left(C_{n}\right)= & 2n\{l_{pn}\left(\hat{\theta }\right)-\max_{\theta ,C_{n}\theta =0}l_{pn}\left(\theta \right)\}\\ = & 2n\{l_{pn}\left({\overset{ˇ}{\lambda }}^{\mathrm{PET}},{\hat{\theta }}^{\mathrm{PET}}\right)-l_{pn}\left(\overset{˘}{\lambda },\overset{˘}{\theta }\right)\}\\ = & nP_{n}\left[G^{⊤}\left(\theta \right)\right]K_{1}^{-1/2}\left(S_{1}-S_{2}\right)K_{1}^{-1/2}P_{n}\left[G\left(\theta \right)\right]+o_{p}\left(1\right), \end{array}$$

where $S_{1}=K_{1}^{-1/2}K_{2}K_{3}K_{2}^{⊤}K_{1}^{-1/2}$ and $S_{2}=K_{1}^{-1/2}K_{2}\Gamma_{1}K_{2}^{⊤}K_{1}^{-1/2}$ are two symmetric idempotent matrices. Hence, there is a matrix $\tilde{S}$, such that $S_{1}-S_{2}={\tilde{S}}^{⊤}\tilde{S}$ and $\tilde{S}{\tilde{S}}^{⊤}=I_{e}$. By the center limit theorem, we obtain that $\sqrt{n}\tilde{S}K_{1}^{-1/2}P_{n}\left[G^{⊤}\left(\theta \right)\right]\overset{L}{⟶}N\left(0,I_{e}\right)$, which leads to $nP_{n}\left[G^{⊤}\left(\theta \right)\right]K_{1}^{-1/2}\left(S_{1}-S_{2}\right)K_{1}^{-1/2}P_{n}\left[G\left(\theta \right)\right]\overset{L}{⟶}\rho_{1}\chi_{1}^{2}+...+\rho_{e}\chi_{1}^{2}$, where the weights $\rho_{1}\leq ...\leq \rho_{e}$ are the eigenvalues of matrix $\left(S_{1}-S_{2}\right)K_{1}^{-1}$. Let $\tilde{M}=K_{1}{\left(S_{1}-S_{2}\right)}^{-1}$; taking the transformation to the constrained penalized ET likelihood function yields $\tilde{l}\left(C_{n}\right)\tilde{M}\overset{L}{⟶}\chi_{e}^{2}$. This completes our proof. □

## References

[1] Godambe V.P. (1991). Estimating Functions.

[2] Carroll R., Ruppert D., Stefanski L., Crainiceanu C. (2006). Nonlinear Measurement Error Models, A Modern Perspective.

[3] Hardin J.W., Hilbe J.M. (2003). Generalized Estimating Equations.

[4] Hansen L.P. (1982). Large Sample Properties of Generalized Method of Moments Estimators. Econometrica.

[5] Kitamura Y. (1997). Empirical likelihood methods with weakly dependent processes. Ann. Stat..

[6] Owen A.B. (1988). Empirical likelihood ratio confidence intervals for a single functional. Biometrika.

[7] Qin J., Lawless J. (1994). Empirical likelihood and general estimating Equations. Ann. Stat..

[8] Tang N., Yan X., Zhao P. (2018). Exponentially tilted likelihood inference on growing dimensional unconditional moment models. J. Econom..

[9] Newey W., Smith R.J. (2003). Higher order properties of GMM and generalized empirical likelihood estimators. Econometrica.

[10] Anatolyev S. (2005). GMM, GEL, serial correlation, and asymptotic bias. Econometrica.

[11] Tibshirani R. (1996). Regression shrinkage and selection via the lasso. J. R. Stat. Soc. Ser. b-Methodol..

[12] Zou H. (2006). The adaptive lasso and its oracle properties. J. Am. Stat. Assoc..

[13] Fan J., Li R. (2001). Variable selection via nonconcave penalized likelihood and its oracle properties. J. Am. Stat. Assoc..

[14] Lv J., Fan Y. (2009). A unified approach to model selection and sparse recovery using regularized least squares. Ann. Stat..

[15] Hastie T., Tibshirani R., Friedman J. (2009). The Elements of Statistical Learning: Data Mining, Inference, and Prediction.

[16] Lam C., Fan J. (2008). Profile-kernel likelihood inference with diverging number of parameters. Ann. Stat..

[17] Zou H., Zhang H.H. (2009). On the adaptive elastic-net with a diverging number of parameters. Ann. Stat..

[18] Caner M., Zhang H.H. (2014). Adaptive elastic net for generalized methods of moments. J. Bus. Econ. Stat..

[19] Wang C.Y., Wang S., Zhao L.-P., Ou S.-T. (1997). Weighted semiparametric estimation in regression analysis with missing covariate data. J. Am. Stat. Assoc..

[20] Wang C.Y., Wang S., Gutierrez R.G., Carroll R.J. (1998). Local linear regression for generalized linear models with missing data. Ann. Stat..

[21] Scharfstein D.O., Rotnitzky A., Robins J.M. (1999). Adjusting for nonignorable drop-out using semiparametric nonresponse models. J. Am. Stat. Assoc..

[22] FitzGerald E.B. (2002). Extended generalized estimating equations for binary familial data with incomplete families. Biometrics.

[23] Wang C.Y., Huang Y., Chao E.C., Jeffcoat M.K. (2008). Expected estimating equations for missing data, measurement error and misclassification, with application to longitudinal nonignorable missing data. Biometrics.

[24] Zhou Y., Liang H. (2008). Statistical inference for semiparametric varying coefficient partially linear models with generated regressors. Ann. Stat..

[25] Tang N., Zhao P., Zhu H.T. (2014). Empirical likelihood for estimating equations with nonignorably missing data. Stat. Sin..

[26] Qi L., Zhang X., Sun Y., Wang L., Zhao Y. (2018). Weighted estimating equations for additive hazards models with missing covariates. Ann. Inst. Stat. Math..

[27] Shao J., Wang L. (2016). Semiparametric inverse propensity weighting for nonignorable missing data. Biometrika.

[28] Tang N., Xia L., Yan X. (2019). Feature screening in ultrahigh-dimensional partially linear models with missing responses at random. Comput. Stat. Data Anal..

[29] Liu T., Yuan X. (2019). Adaptive empirical likelihood estimation with nonignorable nonresponse data. Statistics.

[30] Wang S., Shao J., Kim J.K. (2014). An instrumental variable approach for identification and estimation with nonignorable nonresponse. Stat. Sin..

[31] Stock J.H. (2015). Instrumental variables in statistics and econometrics. International Encyclopedia of the Social & Behavioral Sciences.

[32] Rosenbaum P.R., Rubin D.B. (1983). The central role of the propensity score in observational studies for causal effects. Biometrika.

[33] Imai K., Ratkovic M. (2015). Robust estimation of inverse probability weights for marginal structural models. J. Am. Stat. Assoc..

[34] Robins J., Rotnitzky A., Zhao L.P. (1994). Estimation of regression coefficients when some regressors are not always observed. J. Am. Stat. Assoc..

[35] Robins J., Rotnitzky A., Zhao L.P. (1995). Analysis of semiparametric regression models for repeated outcomes in the presence of missing data. J. Am. Stat. Assoc..

[36] Hirano K., Imbens G., Ridder G. (2003). Efficient estimation of average treatment effects using the estimated propensity score. Econometrica.

[37] Owen A.B. (2001). Empirical Likelihood.

[38] Papini E., Guglielmi R., Bianchini A., Crescenzi A., Taccogna S., Nardi F., Panunzi C., Rinaldi R., Toscano V., Pacella C.M. (2002). Risk of malignancy in nonpalpable thyroid nodules: Predictive value of ultrasound and color-Doppler features. J. Clin. Endocrinol. Metab..

[39] Kim M.K., Park H., Oh Y.L., Shin J.H., Kim T.H., Hahn S.Y. (2024). Role of ultrasound in predicting telomerase reverse transcriptase (TERT) promoter mutation in follicular thyroid carcinoma. Sci. Rep..

[40] Xu T., Gu J., Ye X., Xu S., Wu Y., Shao X., Liu D., Lu W., Hua F., Shi B. (2017). Thyroid nodule sizes influence the diagnostic performance of TIRADS and ultrasound patterns of 2015 ATA guidelines: A multicenter retrospective study. Sci. Rep..

